# Two-action task, testing imitative social learning in kea (*Nestor notabilis*)

**DOI:** 10.1007/s10071-023-01788-9

**Published:** 2023-06-01

**Authors:** Elisabeth Suwandschieff, Amelia Wein, Remco Folkertsma, Thomas Bugnyar, Ludwig Huber, Raoul Schwing

**Affiliations:** 1 Haidlhof research station, Comparative Cognition, Messerli Research Institute, University of Veterinary Medicine Vienna, Vienna, Austria; 2 Platform Bioinformatics and Biostatistics, University of Veterinary Medicine Vienna, Vienna, Austria; 3 Department of Cognitive Biology, University of Vienna, Vienna, Austria

**Keywords:** Kea, Nestor norabilis, Social learning, Imitation, Two-action task, Emulation

## Abstract

Social learning is an adaptive way of dealing with the complexity of life as it reduces the risk of trial-and-error learning. Depending on the type of information acquired, and associations formed, several mechanisms within the larger taxonomy of social learning can be distinguished. Imitation is one such process within this larger taxonomy, it is considered cognitively demanding and is associated with high-fidelity response matching. The present study reproduced a 2002 study conducted by Heyes and Saggerson, which successfully illustrated motor imitation in budgerigars (*Melopsittacus undulatus*). In our study, eighteen kea (*Nestor notabilis*) that observed a trained demonstrator remove a stopper from a test box (1) took less time from hopping on the box to feeding (response duration) in session one and (2) were faster in making a vertical removal response on the stopper once they hopped on the box (removal latency) in session one than non-observing control group individuals. In contrast to the budgerigars (Heyes and Saggerson, Ani Behav. 64:851–859, 2002) the present study could not find evidence of motor imitation in kea. The results do illustrate, however, that there were strong social effects on exploration rates indicating motivational and attentional shifts. Furthermore, the results may suggest a propensity toward emulation in contrast to motor imitation or alternatively selectivity in the application of imitation.

**Supplementary Information:**

The online version contains supplementary material available at 10.1007/s10071-023-01788-9.

## Introduction

Imitation can be defined as the copying of a novel or otherwise improbable act (Thorpe 1963). It is a process within the larger taxonomy of social learning (Whiten et al. 2009) and presents a specific form of social learning, in which an individual acquires a behaviour through “observation of, or interaction with, a conspecific or its products” (Heyes 1994: 207). Social learning is considered an adaptive way of dealing with the complexity of life as it mitigates the risk of trial-and-error learning. Different learning mechanisms, with varying degrees of fidelity–the degree to which the behaviour of the observer matches that of the demonstrator (Whiten and Ham 1992)–can be identified. While in enhancement the individual orients their attention toward a certain object or location (Whiten and Ham 1992) as the primary source of information, in emulation the individual orients their attention towards the result (Tomasello et al. 1989). Imitation combines both levels of abstraction and therefore represents a “high level cognitive achievement” (Whiten and Ham 1992:271) and can be associated with high-fidelity response matching (Huber et al. 2009). For the study at hand, we maintained the definition of imitation used by Heyes and Saggerson “imitation refers to copying by an observer of a feature of the body movement of a model” (Heyes 2001: 254), for a comprehensive listing of social learning mechanisms see Hoppitt and Laland (2013).

Multiple species have illustrated imitative skills in experimental settings, including two-action tasks (Akins and Zentall 1998; Bugnyar and Huber 1997; Voelkl and Huber 2000; Fawcett et al. 2002; Heyes and Saggerson 2002; Nguyen et al. 2005), bi-directional controls (Akins et al. 2002; Heyes et al. 1994; Heyes and Dawson 1994; Mitchell et al. 2018) and Do-as-I-do tasks (Custance et al. 1995; Huber et al. 2009; Fugazza et al. 2015; Tomasello et al. 1993; Call 2001, Topál et al. 2006). However, there are also many studies that failed to provide evidence for imitation (Veit et al. 2023 (*Sus domesticus*); Greco et al. 2013 (*Loxodonta africana africana*); Izawa and Watanabe 2011 (*Corvus macrorhynchos*) to name a view). Although those studies are difficult to explain, it is worth pointing out that they typically do provide evidence for other forms of social learning. For instance, human’s closest relatives the great apes (*Pan troglodytes, Pongo pygmaeus, Pan paniscus*) tend to show low levels of motor imitation, particularly when it comes to high-fidelity copying of actions (Clay and Tennie 2018, Horner and Whiten 2005, Nielson and Susianto 2010). Instead, they tend to use their own actions to produce the demonstrated outcome, i.e. show emulation (Tomasello 1998).

Parrots are large-brained birds and renowned for their capacity to solve complex physical problems, which they achieve both spontaneously and through trial-and-error (Auersperg et al. 2009, 2011, 2012, Dawson and Foss 1965, Funk 2002, Miyata et al. 2011, Pepperberg and Funk 1990, Werdenich and Huber 2006). Yet, despite the recognised physical cognitive abilities of parrots, out of the near 400 parrot species only very few have been tested on their motor imitation capacities (Dawson and Foss 1965, Galef et al. 1986, Moore 1992, Huber et al. 2001, Heyes and Saggerson 2002, Gajdon et al. 2004, Auersperg et al. 2012) and even fewer could successfully demonstrate such behaviour, to date only African greys (*Psittacus erithacus*) and budgerigars (*Melopsittacus undulatus*) have been illustrated to show motor imitation conclusively. Goffin’s cockatoos (*Tanimbar corella*), have illustrated the ability to spontaneously make and use their own tools, and that naïve observers can learn tool-using tasks by watching a trained demonstrator (Auersperg et al. 2012, 2014). However, even though the authors could show strong evidence for emulation, imitation was at best evident at low-fidelity levels (Auersperg et al. 2014). Although the cockatoos only successfully solved the task after watching a demonstrator, they applied different techniques (levering) to achieve this goal. Furthermore, tool manufacturing and use are highly specialized skill sets, which might suggest a specialized predisposition for the advanced social learning which Goffin’s demonstrated.

Heyes and Saggerson on the other hand, tested 28 juvenile budgerigars, which are not known for such specialized technical skills, on imitative and nonimitative social learning using a two-action task. In their test, observer birds saw “a conspecific demonstrator repeatedly remove one of two stoppers from the horizontal surface of a food box, either by pulling the stopper up or by pushing it down into the box” (Heyes and Saggerson 2002: 852). Responses to the demonstrated stopper colours indicate nonimitative social learning whereas responses in line with the extraction topography (pulling vs. pushing) suggest motor imitation (Heyes and Saggerson 2002). Their results illustrated that observer birds preferentially applied the demonstrated opening method and hence engaged in motor imitation.

Kea (*Nestor notabilis*), a large parrot species endemic to the Southern Alps of New Zealand, have well marked technical intelligence (Auersperg et al. 2009, 2011; Gajdon et al. 2004, 2013; Huber et al. 2001; Huber and Gajdon 2006; Miyata et al. 2011; O’Hara et al. 2015, 2016) but have not illustrated motor imitation at the level of other species of the same order (*Psittaciformes*). However, there are only very few studies on the motor imitation capacities of kea. For instance, in 2001 Huber and colleagues tested kea on their ability to “match the response topography or the sequence of (…) [a] model’s actions (movement or sequence imitation)” (Huber et al. 2001: 945). Observer birds watched trained conspecifics open a large steel box by manipulating three locking devices (bolt, split pin and screw) in direct succession. Although observer birds compared to non-observers did show increased “efficiency at unlocking the devices (which) seemed to reflect the acquisition of some functional understanding of the task through observation (emulation learning)” (Huber et al*.*, 2001: 945), they did not engage in motor imitation. This raises the question of whether kea are simply more prone to emulation (Huber et al*.*, 2001), as imitation has not provided a selective advantage in the course of their evolution, or whether the right favourable conditions to test imitation in this species have simply not yet been devised (Huber 2002).

In the study at hand, we, therefore, attempted to further investigate kea’s ability to motor imitate by closely reproducing a study that has successfully illustrated imitation in budgerigars (Heyes and Saggerson 2002). We present a close replication of the methods applied by Heyes and Saggerson, with minor adjustments to the sample size, experimental group composition and materials to account for kea specificities (i.e. neophilia and larger size), by following an analogous experimental protocol. Through reproducing the experimental protocol of a successful study, illustrating motor imitation in *psittacines*, we hoped to set favourable conditions to test motor imitation in kea. Our hypothesis was that watching a conspecific demonstrator solve a two-action-task will affect the action performed by the test subject (observer). We predicted that kea will show increased use of the demonstrated action over alternative actions and that test group individuals will be quicker to approach and gain access to the test box than non-observing control group individuals.

## Methods

### Study site and housing

The kea subjects in this study were permanently housed in a flock of 23 birds in an outdoor aviary (52 m × 10 m × 4 m) at the Haidlhof research station in Bad Vöslau, Austria. The aviary in which they were housed was equipped with sand substrate, hanging branches for perching, shelters, and a variety of enrichment which was renewed regularly. The kea had access to water ad libitum and food was provided three times a day and consisted of fruits, vegetables, seeds, and protein once daily (cooked meat or eggs). Subjects were never food restricted for the purposes of experiments, and all research was non-invasive. In contrast, Heyes and Saggerson maintained the test subjects at 90% bodyweight for the test rotation.

Testing took place in one of two testing compartments (10 m × 6 m × 4 m) in the aviary. These compartments were closed off during testing, but remain open otherwise and are part of the main living area for the kea. All subjects have been previously trained to enter and exit the experimental compartments on verbal command, and all participation was voluntary. Subjects could refuse to participate by ignoring the command to enter the compartment, and they could end sessions early by retreating to perches or shelters, laying down on the ground, or otherwise refusing to participate. If a subject ended a session early, it was directed back into the main aviary and tested again another time.

### Apparatus and set-up

Heyes and Saggerson trained and tested their budgerigars in a holding room in a metal testing cage with all sides occluded by white cardboard (Heyes and Saggerson 2002). The cage was separated into two compartments, i.e. demonstration/test and observation compartment. Comparably, the testing compartment of the kea aviary is a) visually occluded from the rest of the aviary during testing, and b) can be separated into two sub-compartments by closing a mesh wire door in the centre. The two sub-compartments served as the demonstration/test and observation areas. As the kea test compartment is large compared to the testing cage of the budgerigars, two 1 × 1 × 1 m tunnels made out of semi-opaque (milky) plastic, were added to simulate the testing cage used with the budgerigars. The test cage and tunnels decrease the space available to the individuals and thereby likely funnel and channel the view (via the semi-opaque side walls) and guiding the attention towards the demonstration area by reducing environmental (visual) noise.

**Fig. 1:**
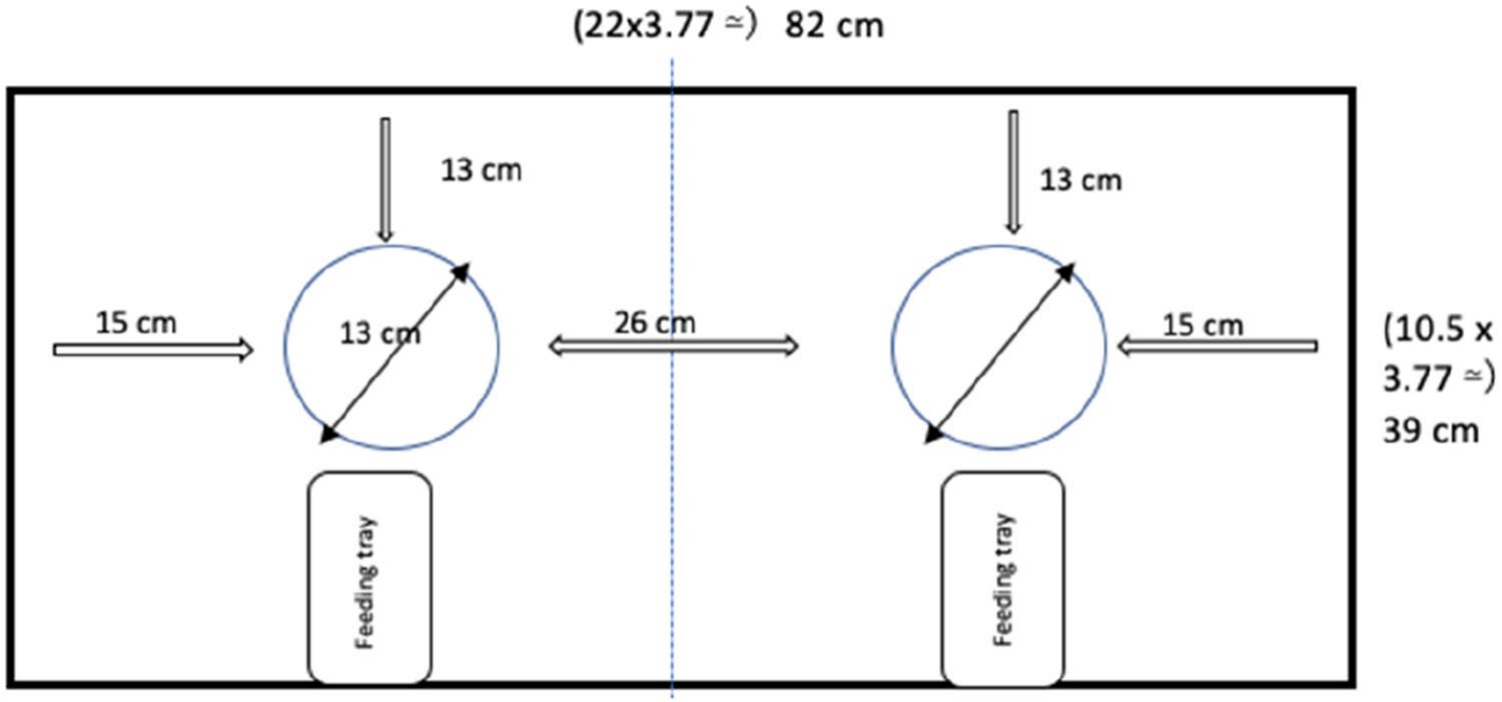
Kea test box specification, including feeding trays, and removable lid. Adjusted by a factor of 3.8 (incl. test box, feeding holes and stoppers) to the budgerigar test set-up to account for the larger size of kea

As a ‘test box’ Heyes and Saggerson used a white, rectangular box (22 × 10.5 × 4.5 cm). The box was divided into equally sized sections and two holes in a white lid provided access to the rewards in the sections below. We adjusted the measurements of the budgerigar test box by a factor of 3.8 to account for the larger size of kea (who are on average 3.8 times longer than budgerigars), resulting in a comparable rectangular wooden test box (see Fig. 1). As in the budgerigar study, the lid of the box featured two feeding holes (also adapted by a factor of 3.8) and the box was divided in half to create two feeding sections. In contrast to the budgerigars, who received time-based millet seed rewarding, kea were rewarded with access to ¼ piece of peanut–their preferred reward that is routinely used–and therefore, additional reward trays were integrated inside the box (see Fig. 1). These reward trays were added to mimic the direct availability of the reward upon opening the box and to prevent the kea from having to search the box for a single piece of peanut.

**Fig. 2:**
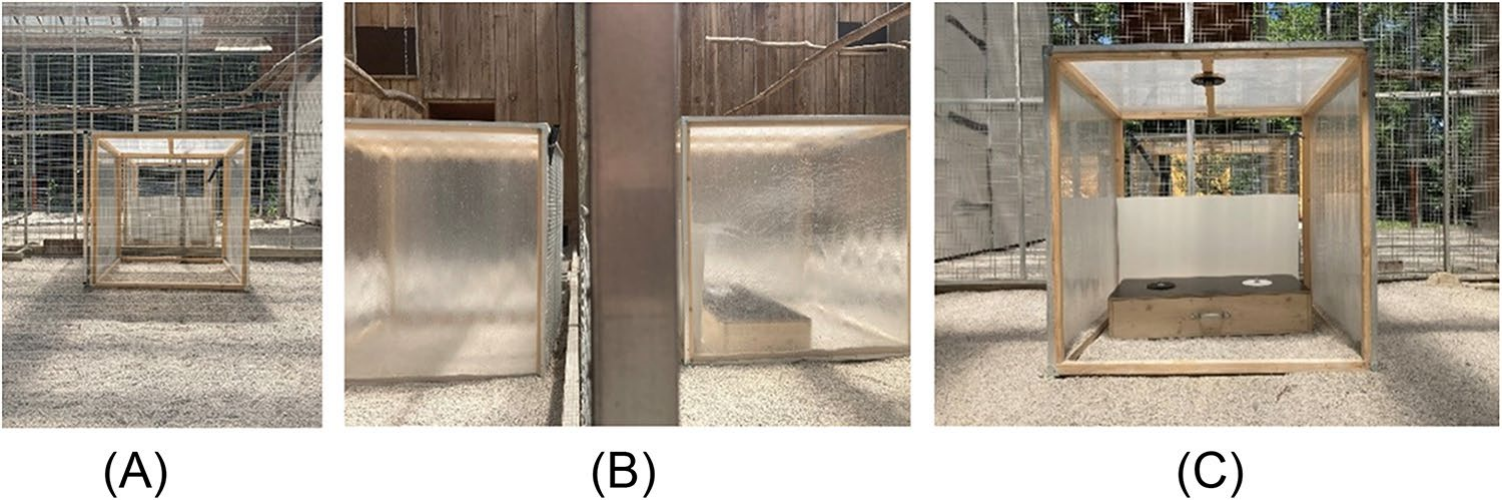
Kea test cage set-up: **A** observation compartment view, **B** two tunnels up against mesh wire separation and **C** demonstration/test compartment view including test box with stoppers and removable visual barrier

As in Heyes and Saggerson, the test box was placed on the floor at the far end of the tunnel, directly up against the mesh wire, with its long axis facing the mesh wire. The second tunnel was placed directly opposite the test box on the other side of the mesh wire (see Fig. 2).

### Stoppers

As stoppers for their box, Heyes and Saggerson used two ping-pong balls cut in half. The open half of the cut ping-pong ball had wire crossed over the top, to allow the birds to grip it. The other side had a smooth surface with no further alteration. The exposed sides of both stoppers were coloured in royal blue or black. Additionally, “the rim of each hole (for the stoppers) was lined with a thin (2 mm) strip of sponge” (Heyes and Saggerson 2002: 853) to provide friction material for the stoppers to stay in place. They used metal brackets, attached to the upper or lower rim of the stopper to prevent all but one removal response on the stoppers in training.

**Fig. 3:**
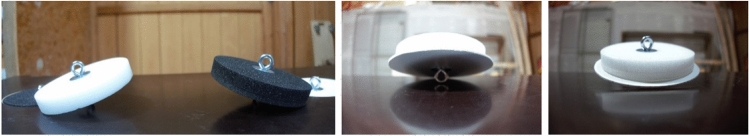
Test stoppers: on top and inside the test box; training stoppers with integrated discs from different angles (below and above)

In our set-up, we also provided the kea with circular stoppers of two different colours. In contrast to the budgerigars, the stoppers for the kea were made of durable foam cut into 13 cm diameter disks with a width of 2 cm (see Fig. 3). These stoppers better matched the keas’ large size and also had the advantage that they stayed set in the feeding holes without additional friction material. Kea are highly inquisitive, and any additional material in the holes could have been a distraction. A loop that matched the keas’ beak size was attached to the exposed side of the stopper, which they could use as a grip. Finally, as in the budgerigar study, two different stopper colours were used, but to make the difference more salient these were black and white, rather than black and royal blue.

For demonstrator training the stoppers were equipped with an additional 17 cm plastic disc attached to the bottom part of the stopper (see Fig. 3). The wider part of the disc was placed inside the hole. This prevented the pulling response and ensured that demonstrators could only solve the task by pushing (see explanation below in the Subjects section). Additional brackets inside the box ensured that only the correct stopper could be removed. The training stoppers were also used in the demonstration sessions to ensure that demonstrations were always solved correctly. Both training and testing stoppers looked identical from the top, making it impossible for the observers to differentiate them on a visual basis.

### Subjects

Eighteen of the 23 kea were available to participate in the study (Table 1). Subjects were 9 females and 9 males and ranged in age from four to 22 years old.

**Table 1:** Test subject identification (ID), sex, age, rearing method (hand vs. parent), group number, experimental group assigned, colour assigned in bold for demonstrated colours (demonstrators only) and italic for observed colours (test groups only) and demonstrator assigned (only applicable for test groups)

	ID	Sex	Age	Raised	Group number	Experimental group	Colour	Demonstrator
1	Fr	♂	17	Parent	1	Control CG1	*Black*	NA
2	Jo	♂	22	Parent	1	Control CG1	NA	NA
3	Pa	♂	11	Parent	1	Control CG1	*White*	NA
4	Ro	♂	13	Parent	1	Control CG1	*White*	NA
5	Fy	♀	5	Parent	2	Control CG2	NA	NA
6	Sy	♀	14	Hand	2	Control CG2	NA	NA
7	Co	♀	14	Hand	3	Test TG1	*White*	Ro
8	Je	♂	6	Hand	3	Test TG1	*White*	Ro
9	Pl	♀	14	Hand	3	Test TG1	*Black*	Fr
10	Pn	♂	4	Hand	3	Test TG1	*White*	Pa
11	Sk	♂	4	Hand	3	Test TG1	*Black*	Fr
12	Di	♀	4	Hand	4	Test TG2	*Black*	Fr
13	Ke	♂	17	Hand	4	Test TG2	*White*	Ro
14	Pi	♂	17	Hand	4	Test TG2	*Black*	Fr
15	Pu	♀	8	Hand	4	Test TG2	*White*	Pa
16	Ly	♀	14	Hand	5	Control CG3	NA	NA
17	Ti	♀	3	Parent	5	Control CG3	NA	NA
18	Wy	♀	14	Hand	5	Control CG3	NA	NA

There were several important differences in our study design compared to the one we were replicating. First was the number of subjects. Heyes and Saggerson used 36 juvenile budgerigars, eight as demonstrators, and 28 as observers. The kea population at the research station Haidlhof is smaller and therefore fewer individuals were available for testing. We also introduced a control group, with an equivalent sample size to the test group (*N* = 9), which was not present in the Heyes and Saggerson study. This control group was necessary because, based on extensive experience working with the kea, we predicted that they were much more likely to pull the stoppers out of the experimental box, rather than push them. If this were the case, then there would be no need for a test group that observed the “pull” action, as we predicted that kea would solve the task through trial-and-error by pulling. On the other hand, we predicted that “push” would be a much more improbable action for kea. Therefore, we introduced Control Group 1 (CG1, *N* = 4)) to test for this. The results of CG1 confirmed our prediction that kea preferentially pull, rather than push, as all subjects pulled the stoppers in every trial, with no instances of pushing. Based on these results, demonstrators were trained only on the “push” action.

The subjects in CG1 were chosen with the intent of re-training them to serve as demonstrators for the test groups. As there was no control group in the Heyes and Saggerson study, this was also a difference in their experimental design. We purposely chose the four highest-ranking adult males in the kea group (Fr, Jo, Pa, Ro), to create as homogenous a group of demonstrators as possible. After completing the control sessions, Jo dropped out of the experiment due to breeding. The remaining three demonstrators (Fr, Pa, Ro) were re-trained on the “push” action. In addition, Pa and Ro were trained that only the white stopper was baited, and Fr was trained that only the black stopper was baited.

Additional control groups (Control Group 2 (CG2, *N* = 2) and 3 (CG3, *N* = 3)) were tested later to create a balanced comparison between the test groups and control groups. Due to availability of individuals (breeding season) these groups had to be split in two (CG2 and CG 3 respectively) and were tested at different times. Control subjects were chosen according to the following criteria: during test sessions, observers had to remain in an observation tunnel for the duration of the demonstration (see the section on Apparatus and setup), but some subjects were hesitant to enter or remain in the tunnels despite extensive training. Therefore, those individuals that could not be trained to remain inside the tunnel were assigned to the control groups. In total, we had three Control Groups (CG) comprising nine individuals.

All individuals that could be trained to enter and remain in the tunnels during demonstration sessions were included in the test groups. Test Group 1 (TG1) was comprised of five individuals and Test Group 2 (TG2) of four individuals, for a total of nine test individuals (see Table 1 for further details).

### Habituation and training procedure

As with the budgerigars, the kea were trained and tested in the testing compartment. First, they were target-trained to enter the observation tunnel, and as mentioned above, the subjects who failed this training were assigned to the control groups. All individuals were test box trained, regardless of the experimental group. In test box training, subjects were separated individually into the testing compartment and were allowed to feed from the test box inside the tunnel, or outside for the control group birds which did not tolerate the tunnels. The stoppers were absent during test box training, and both sides were rewarded. Following the budgerigar protocol, once an individual successfully fed from the test box in 100% of trials in 3 consecutive sessions of 10 trials each, their training was considered complete. On average CG individuals spent 3.9 and TG individuals 5.2 sessions in test box training. Feeding bout reduction sessions were not necessary for the kea as they received access to a single piece of peanut. Due to keas’ neophilic nature, no habituation to the stoppers was necessary or conducted.

Following Heyes and Saggerson the demonstrators received additional stopper training, where the training stoppers were fixed to prevent all but one response. This training was more complex and involved four phases, each with its own criterion for advancement (10/10 correct in two consecutive sessions, with no wrong side attempts), for the detailed training protocol see Online Resource 3. Once the demonstrators had successfully reached criterion in all four phases they were ready to perform their demonstration. During testing none of the demonstrators deviated from their assigned opening method or colour.

### Testing procedure

Heyes and Saggerson tested two repetitions of the two-action task, with the first repetition using constant stopper positions across trials (blue and black always on the same side) and the second repetition using randomised colour/side assignment of the stoppers. In our current replication study, we tested the kea *only* on the second repetition, so that the side on which the positive stimulus occurred was randomized from trial to trial. Also note that, while the Heyes and Saggerson experiment consisted of four test conditions (black pull, black push, blue pull, blue push), the kea had two test conditions (black push, white push) and one control condition with no demonstration.

Following the test session protocol of Heyes and Saggerson, each CG individual received seven test sessions at 10 trials each over four consecutive days, with sessions taking place in both the morning and afternoon. CG birds that were called into the testing compartment were allowed to approach the test box and manipulate it based on trial and error. All responses were rewarded regardless of colour choice or method. Once an individual chose one response and retrieved their reward, it was called back to the observation compartment. The test box was rebaited, and the stoppers were replaced based on randomised colour-side combinations.

The testing schedule for the kea was exactly the same as that for the budgerigars. Test subjects received ten sessions altogether, one in the morning and one in the afternoon, over five consecutive days. Each session started with the demonstrator in the demonstration compartment with the test box and the test subject (henceforth observer) in the observation compartment. The first three sessions were demonstration-only, in which the observers saw their respective demonstrator open the test box ten times in a row. Starting in the fourth session and continuing to the tenth and final one, observers were given test sessions directly after viewing a demonstration session, in which they gained access to the experimental box. These demonstration/test sessions started with ten demonstration trials, after which the demonstrator was removed from the testing compartment. The observers were then given access to the box for ten consecutive trials, with no other individuals present.

All responses were rewarded for the observers, regardless of colour choice or method. Once a subject gained access to the reward by removing one of the two stoppers, the trial was ended and the subject was called back to the observation compartment. The test box was re-baited and the stoppers replaced based on randomised colour/side combinations.

In contrast to the budgerigars, kea were unrestricted in their movement within the larger demonstration and observation compartments, as they were free to enter and exit the tunnels. To ensure that the observers were present inside the tunnel during the demonstration trials, Experimenter 1 directed the subject inside using target training before each trial began. Once the observer was inside the tunnel, Experimenter 2 gave the demonstrator access to the tunnel with the test box inside. After the demonstrator removed the stopper and retrieved the reward, Experimenter 2 directed him out of the tunnel and re-baited the box for the next trial. For the test sessions, where the demonstrator was not present, observers were given access to the test box when Experimenter 1 opened the door to the demonstration compartment, and they were sent back to the observation compartment using a verbal command when they had solved the task.

Each individual (test and control group) had unlimited time to engage with the task, unless, an individual left the testing compartment or did not engage with the task for more than 15 min the session was terminated. This was the case in four sessions, for three individuals. For examples of demonstration session and test sessions see the videos provided in the supplementary materials (Online Resources). All experimenters present in the test compartment wore mirrored sunglasses in all demonstration and test sessions so that they would not provide any inadvertent cues to the participating subjects.

### Data scoring

All experiments were videotaped from two sides, behind the observation compartment and directly above the test box within the test compartment tunnel. All recordings of the test sessions were scored/coded (excluding the observation-only sessions) with Solomon Coder (version beta 19.08.02). Following our hypothesis and predictions the succeeding behaviours were coded for all test sessions: 1) the approach duration from the opening of the gate to hopping onto the test box with both feet touching (to estimate how fast individuals were in approaching the box), which was converted to the approach pace, see below; 2) the removal latency from landing on a test box with both feet to the opening method (vertical movement) with the stopper being pulled out or pushed into the box (to estimate how long it took individuals to make a response); 3) the total response duration from landing on the test box to feeding, hence, completing the trial by retrieving the reward (to estimate how long it took individuals to solve the task); as well as 4) the response type frequency of push vs. pull, black vs. white (to estimate how many individuals used the respective opening method). As the TG individuals were tested in the tunnel and the CG individuals were not, the distance to approach the test box was different for the two experimental groups. Therefore, the approach pace was calculated for each individual and each trial by dividing the approach time by the approach distance. The resulting pace made the individual traveling times comparable between groups irrespective of distance. One independent rater, who was blind to the study, scored 10% of all videos (assigned at random) to check for the interobserver reliability, with Kappa for the Categorical (push versus pull *k* = 1.00) and Intraclass Correlation Coefficient for the numerical data (approach duration ICC = 0.99, removal latency ICC = 0.99, response duration ICC = 0.94).

### Statistical analysis

To analyse the behaviour of the Kea several generalized linear mixed models (GLMM) were fitted in R (version 4.2.0; R Core Team, 2021) using the function lmer of the package lme4 (version 1.1–27; Bates et al. 2015), using data from all completed trials. To answer our main research question–whether Kea matched the behaviour of the demonstrators–we used a binomial GLMM to test if the probability to push differed between experimental groups. To control for potential colour or side (bias) effects we ran a binomial GLMM to test whether there was a difference between the experimental groups concerning colour choice (white *versus* black) and side (left *versus* right). To assess whether the time it takes to complete the task differed between experimental groups we ran three linear mixed models. First, using the pace to approach the box as a response variable, we tested whether the observer birds were faster to approach the test box. With a second model, we investigate whether the total time to complete the task differed between experimental groups. Finally, we tested whether the latency to make a removal response differed between experimental groups, for details on the analysis see Online Resource 1. In all of the above models our key fixed effects were experimental group, session number, trial number, and all their interactions up to the third order. Additional fixed effects were age and sex. We also included random intercepts for the individual and sessionID (a factor combining session number and individual) nested within the individual. We included random slopes of session number and trial number and their interaction in individual as well as the random slope of trial number in sessionID. Full models were compared to their respective null models excluding the key fixed effects of interest (experimental group; session number; trial number) and all their interactions up to the third order, but otherwise similar with respect to the random effects structure to avoid cryptic multiple testing (Forstmeier and Schielzeth 2011). Cases in which an individual was carried in were excluded (on a trial basis) from approach pace and response duration (four individuals in six sessions and altogether nineteen trials) additionally we also excluded non- participation cases (three individuals did not participate in one session and one individual did not participate in four sessions). Our main models included data from all sessions (session 1–7) to make it comparable to Heyes and Saggerson. However, as kea are very fast learners, we predicted that potentially large differences between experimental groups would be most pronounced in the first session and may be gone by session two and onwards. A linear model may have little power to detect such a non-linear effect, therefore, session 1 was tested separately. To contrast our results from session 1 to the remaining sessions we also analysed sessions 2–7, see supporting material (Online Resource 1 and 2) for further information on model details, output and model diagnostics.

## Results

Fourteen of the 18 individuals that participated in testing completed all seven test sessions with all ten trials. From the four individuals that did not complete all sessions and trials, two individuals completed six sessions (ID7 and ID18), one individual completed five sessions (ID 8) and the first three trials of session six and one individual completed three sessions (ID9), for details on IDs see Table 1. With regard to stopper colour choice, the full-null model comparison of the linear mixed effects model illustrated that there was an interaction between the experimental group, trial and session (*χ*^*2*^ = 5.285, df = 1, *P* = 0.022), revealing that while the control group was more constant in their selection behaviour the test group had a slightly higher probability to choose white, especially in later sessions and trials (see Online Resource 2). However, there was no significant difference between experimental groups concerning colour choice overall (*χ*^*2*^ = 2.041, df = 1, *P* = 0.15, see Online Resource 1 Fig. 6)*.* In addition, all groups illustrated a strong side bias to the side that was closest to them (left side), however, this bias was the same across experimental groups (*χ*^*2*^ = 0.13, df = 1, *P* = 0.72, see Online Resource 1 Fig. 7).

### Removal response

The predictors, experimental group, session number, trial number and/or their interaction, did not have an effect on the removal response (i.e., pushing or pulling stopper), as indicated by the full-null model comparison (*χ*^*2*^ = 6.630, df = 7, *P* = 0.468). The main response for both groups was to pull (control group *N* = 593, test group *N* = 555). Push did occur on 16 separate occasions across experimental groups with no significant difference between the groups concerning the frequency (control group *N* = 10, test group *N* = 6), emphasising that their natural response is to pull. With respect to the response to push or pull there was no significant difference between experimental groups (chi-square test, *χ*^*2*^ = 1.167, df = 1, *P* = 0.280). Observer birds did not align their removal response with the demonstrated direction/method.

### Approach pace

Overall, the test predictors did not have an impact on the approach pace (full-null model comparison for all sessions (likelihood ratio test, *χ*^*2*^ = 9.775, df = 7, *P* = 0.202)). Observer birds' average approach pace did not differ from the control group (observers: 2.82 (1.68) mean (SD); control: (3.68 (7.87)). The model revealed considerable variation among individuals as indicated by the estimated contribution of the random effect of individuals being in the same order of magnitude as the fixed effects. For instance, the estimated fixed effect of the experimental group was 0.216, while the random intercept of individuals contributed a standard deviation of 0.338, indicating interindividual variation was large compared to the difference between experimental groups. Individual full-null model comparisons for session 1 and session 2–7 did not reveal any effects of the test predictors on approach pace, see Online Resource 2.

### Response duration

Concerning the time it took individuals to complete the entire task (response duration: from landing on the box to feeding) the full-null model comparison revealed that overall the test predictors had an impact (*χ*^*2*^ = 24.448, df = 7, *P* = 0.0010) on the outcome variable. However, controlling for removal latency within response duration over all sessions illustrated that there was no more clear effect between the experimental group, trial, session or any of their interactions (*χ*^*2*^ = 4.587, df = 7, *P* = 0.71), see Online Resource 1 Fig. 9.

Further analysis of session 1 (while controlling for removal latency) revealed a marginally significant effect of the test predictors as a whole on response duration (*χ*^*2*^ = 6.808, df = 3, *P* = 0.078). Looking closer at this we found a significant interaction between the experimental group and trial number (*χ*^*2*^ = 6.105, df = 1, *P* = 0.014). This means, while taking the effect of removal latency into account, the response duration of the control group slightly increased, whereas observer birds decreased in response duration over the trial number (see Online Resource 2). However, the influence of the test predictors was no longer detectable by session 2 (*χ*^*2*^ = 6.641, df = 7, *P* = 0.47).

### Removal latency

**Fig. 4:**
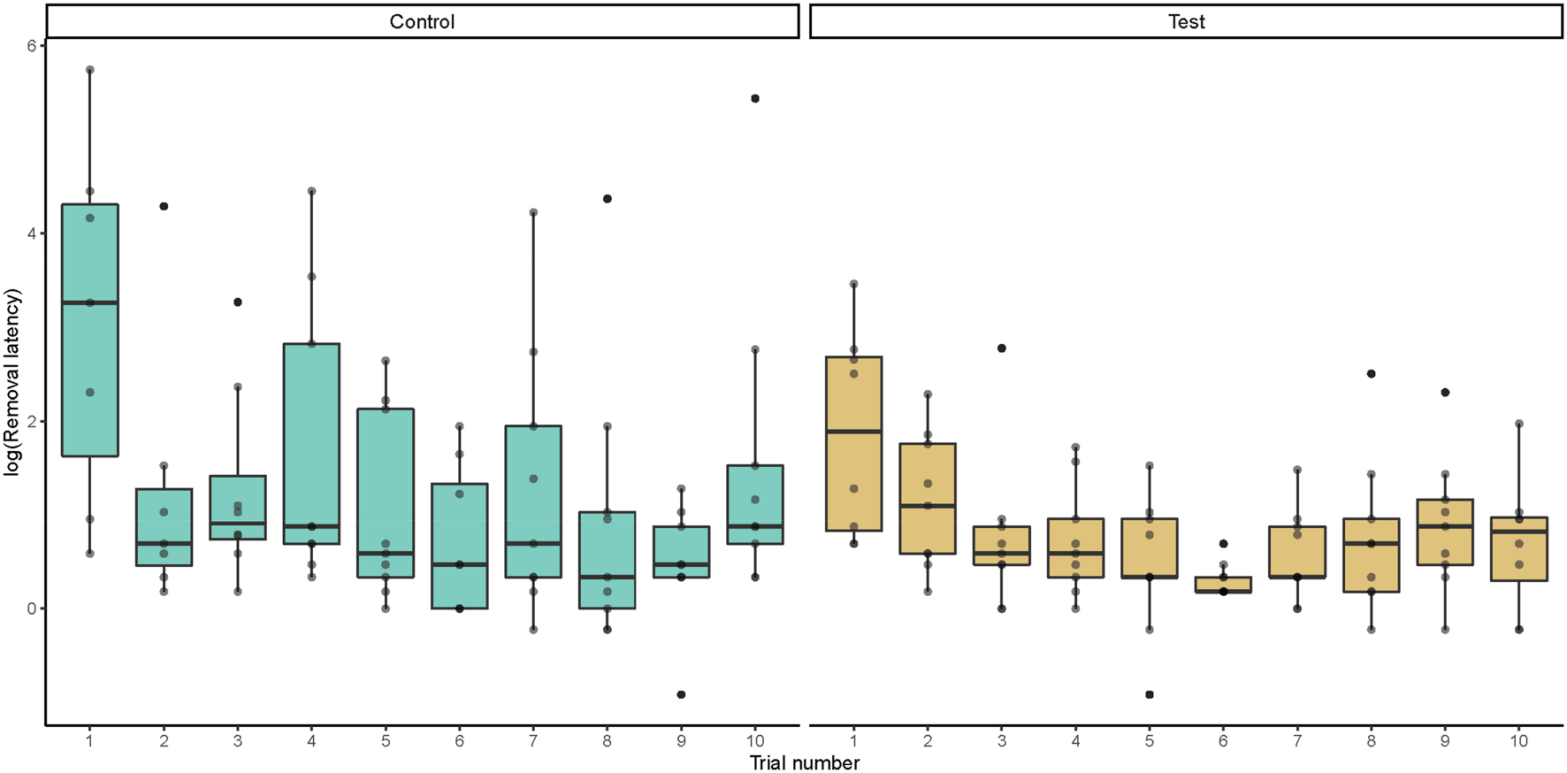
Log of the removal latency session 1 for control (green) and test (yellow) birds across trials in the first session. Boxplots show the median (solid line), 25th–75th percentile (box) and the largest and smallest value (whiskers). Dots reflects outliers. Note that the figures show the raw data and does not represent the fitted model with log (removal latency) as a response. Full-null model comparison: χ.^2^ = 21.579, df = 3, P = 0.000079

The latency to make a removal response was influenced by the test predictors (*χ2* = 29.775, df = 7, *P* = 0.0001), but the three-way interaction between test predictors was non-significant. After removing this and other non-significant interaction terms we found effects of the interaction between session and trial. The removal duration showed a strong decrease over successive trials in session 1 (see Fig. 4), but did not change much in the later sessions, see Online Resource 1 Fig. 10. On average the observer birds took a similar amount of time to remove one of the two stoppers than control group birds did.

As the main effect was expected in the first session with individual learning occurring in all groups once the task was acquired we ran a separate analysis for session 1 and session 2–7, respectively. And indeed, after subsetting the data to only contain session 1, the full-null model comparison revealed an effect of the test predictors (*χ*^*2*^ = 21.579, df = 3, *P* = 0.000079), but revealed no significant interaction between the experimental group and trial. After removing the interaction, the effects of the experimental group (*χ*^*2*^ = 7.167, df = 1, *P* = 0.0074), trial (*χ*^*2*^ = 12.217, df = 1, *P* = 0.00047) and age (*χ*^*2*^ = 5.923, df = 1, *P* = 0.015) all appeared significant, see Online Resource 2. The observer birds (test group) on average took 0.745 log seconds less to make a removal response in session one and older birds were faster than younger birds.

There was an effect of trial which did not differ between experimental groups as well as an average decrease of latency across all groups to remove a stopper. Birds were on average 0.328 log seconds faster per trial to remove stoppers. The full-null comparison for the subset including sessions two to seven did not reveal any effect of the test predictors (*χ*^*2*^ = 11.108, df = 7, *P* = 0.134).

### Sex differences

**Fig. 5:**
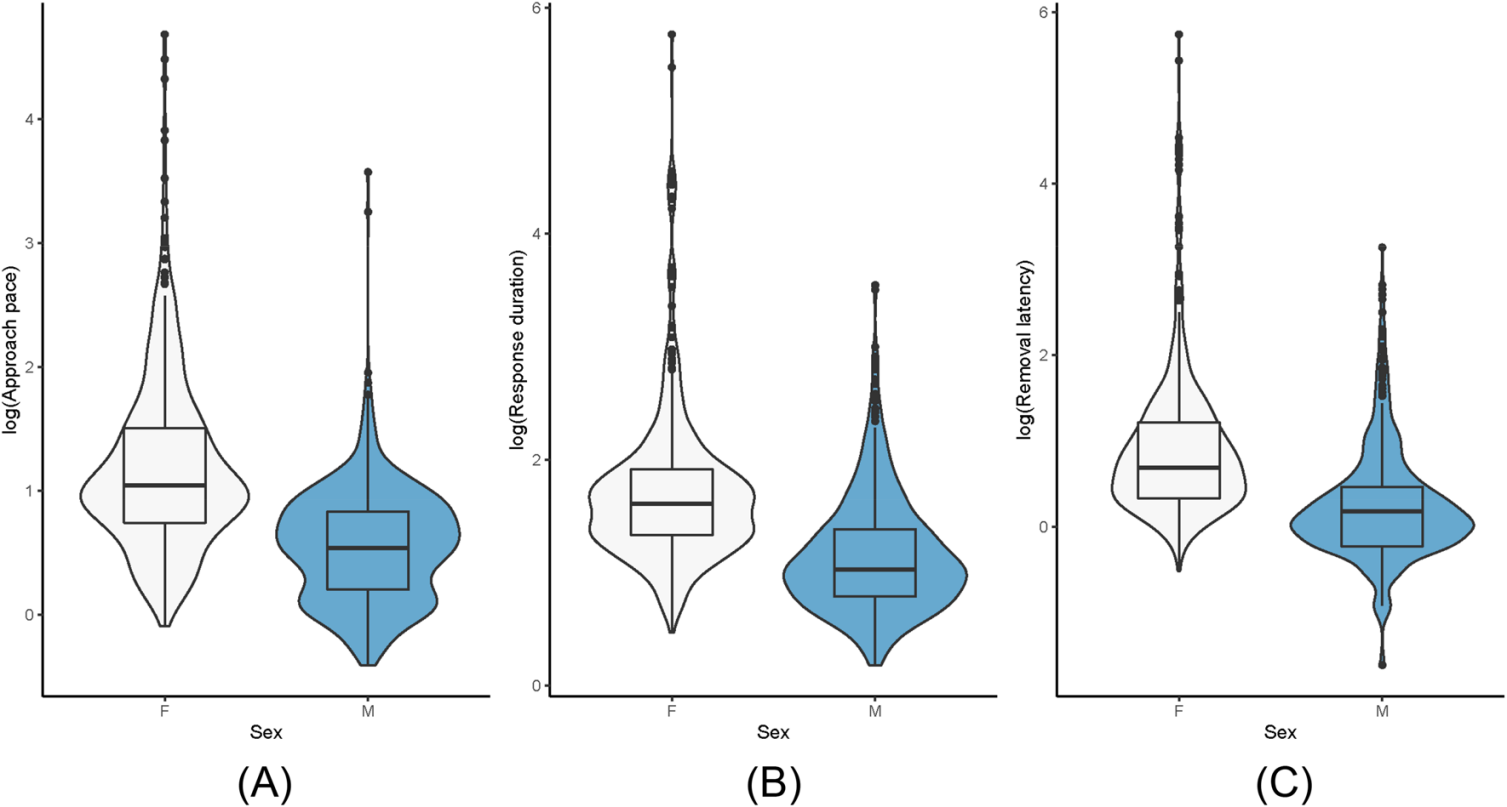
Logs of the approach pace, response duration and removal latency for females and males across all trials and sessions. Sex differences in **A** approach pace between females (F) and males (M), *P* = 0.0005; **B** in response duration between females (F) and males (M), *P* = 0.0119 and **C** removal latency between females (F) and males (M), *P* = 0.0079. Violin plots with boxplots showing the median (solid line), 25th–75th percentile (box) and the largest and smallest value (whiskers). Dots reflects outliers. Note that the figures show the raw data and not the corresponding models

Each of the above models also included sex as a control predictor; the analysis of the individual models revealed that there was a significant difference between the sexes in approach pace, response duration and removal latency across all trials and sessions. Males on average were significantly faster in approaching the test box (males versus females: 1.99 (1.89) versus 4.72 (7.99) seconds, (*χ*^2^ = 12.198, df = 1, *P* = 0.0005); males had a shorter response duration to complete the task (males versus females: 2.0372 (1.64) versus 3.04 (3.56) seconds; (*χ*^2^ = 6.321, df = 1, *P* = 0.0119)); and males were faster to make a removal response (males versus females: 1.69 (2.06) versus 5.27 (19.1) seconds; *χ*^2^ = 7.049, df = 1, *P* = 0.0079)), see Fig. 5.

## Discussion

Kea that observed a trained demonstrator (1) took less time from hopping on the box to feeding (response duration) in session one, and (2) were faster in making a vertical removal response on the stopper once they hopped on the box (removal latency) in session one than non-observing control group individuals. Despite the clear social effects on observers’ initial behaviours, there was no matching of the demonstrated stopper colour or action. Hence, in contrast to budgerigars’ (Heyes and Saggerson 2002), the present study could not find evidence of motor imitation in kea.

The highly significant effect of the experimental group, in regards to removal latency in session one, suggests that observers extracted some information from the demonstration. On the motivational level (Zentall 2006), the mere presence of a conspecific could have amplified the motivation to participate in the task (social facilitation, Zajonc and Sales 1966). Hence, the demonstration might have incentivised observers to engage with the set-up and led observer birds to be quicker to respond than the non-observing control. On a perceptual level, the demonstration could have also amplified the spatial location of the stimuli test box and stoppers via the process of local enhancement (Thorpe 1963). Observer birds, therefore, had the benefit of knowing the relevant locations of the test set-up and could exploit this advantage in their first attempts at solving the task.

The response duration decreased across trials in session one for observer birds while it increased slightly for the control group, pointing towards a demonstration effect. Conversely, control group individuals illustrated a steep decline in response duration in session two, allowing them to catch-up by the third trial of the second session and redressing any differences between the groups. The demonstration had no more effect once an individual had gained experience at solving the task, erasing any differences between the groups by session two. In contrast, the budgerigars increased their response matching in a linear fashion across the seven test sessions indicating persistent demonstration effects (Heyes and Saggerson 2002). For kea, the individual experience seemed to trump any advantage an individual could gain through a demonstration, eliminating any significant difference between the groups. This is in line with previous findings from Huber and colleagues (2001) who illustrated that for kea individual experience with a task takes precedence over close copying of a conspecific demonstrator. It is likely that kea abandon paying any attention to a demonstration once they have acquired the necessary information to solve the task on their own. However, the activities of the demonstrator did enhance the performance of the observers significantly in the first session, with the observer birds being faster to remove the stoppers in their first attempts. This points towards kea possibly having learned something about the relationship between a given stimulus and the reinforcement which amplified their motivation. In other words, the observed connection between manipulandum and reinforcement for the demonstrator could form a “Pavlovian association” (Whiten and Ham 1992) for the observer. This association between stimulus and reinforcement would indicate observational conditioning (Zentall 2006). These results are in line with previous studies on kea illustrating that motivation, manipulation propensity and efficiency are amplified by a demonstration (Huber et al*.*, 2001), albeit without including imitation effects.

Arguably, the combination of social facilitation, local enhancement and observational conditioning is an efficient way of dealing with foraging problems. These mechanisms mitigate the risk of trial-and-error learning that is solely reliant on spatial or environmental cues. By responding to conspecifics with increased motivation (social facilitation), focusing the attention on the box (local enhancement), with the anticipation of a reward (observational conditioning) individuals will likely succeed in solving a task. If we assume that kea gather as much information as possible (about place, object and potential reward) from observing a knowledgeable demonstrator, the question remains why they favour individual strategies of solving the task over imitation, especially in the first trial/session. In the first instance, a demonstration shows the direct pathway from manipulation to reward, it would, therefore, seem beneficial for any observer to follow said pathway to obtain the reward. Our results, however, illustrated that even though all birds successfully solved the task, none of the observers applied the demonstrated opening method to achieve this goal. Even with a propensity for individual manipulation, in the first instance, imitation would constitute the most promising strategy. It, therefore, seems that an additional level of kea behaviour has to be considered when discussing a lack of imitation.

All kea were likely to engage with the task due to their highly neophilic and exploratory nature. Their exploratory characteristic corresponds with their natural feeding strategies, as extractive and opportunistic group foragers (Diamond and Bond 1999) and has likely promoted social learning. Indeed, kea fulfil many criteria that may promote social learning, such as long lifespan, several reproductive cycles, extended juvenile periods and care, group foraging, curiosity and resourcefulness (Lefebvre 2000; Richardson and Boyd 2000 in Gajdon et al. 2004, 2006). Accordingly, experimental evidence for social learning in kea has been provided by Huber and colleagues (2001). Furthermore, field observations have shown that kea pay close attention to what conspecifics are feeding on and interacting with (Diamond and Bond 1999). However, we know from juvenile kea that they are not prone to directly reproducing specific foraging techniques, in terms of action patterns or topographies, but rather focus on obtaining information about the appearance and location of any potential food resources (Diamond and Bond 1999). Within the process they learn to “recognize and locate food, the social structure [however] reduces the incentive and opportunity for them to practice individual foraging” (Diamond and Bond 1999: 98). It is therefore very probable that even as adults kea pay attention to the information provided by others concerning the location and quality of resources while at the same time depending on individual strategies to manipulate and extract said resources.

In general, island species, as kea are, have been shown to exhibit more exploratory behaviour, hence spending more time acquiring information on individual resources than mainland species. For instance, in 2002 Mettke-Hofmann and colleagues tested 76 species of captive parrots (*Psittaciformes*) on their exploratory behaviour and found that there was a significant difference in exploration time between island and mainland species with the former spending more time on exploratory behaviour. They argue that “the value of information is high on islands and exploration helps island species to acquire information” (Mettke-Hofmann et al. 2002: 267) which is particularly valuable in areas with fluctuating food availability. Similarly, while the number of food sources kea can take advantage of is high, with “as many as a hundred species of plants and animals” (Diamond and Bond 1999:17), there is a relative scarcity of any one resource across their highly diverse habitat. Therefore, the depletion rate of any resource is very high and the likelihood to find that resource at the exact same location is very low. After all, imitation “may also be disadvantageous, if copied information is outdated or mismatched to the observing individual” (Aplin et al. 2017:7837). Thus, for kea it might be advantageous not to waste time on a potentially depleted resource and to gather as much information as possible on how to access that resource at another place.

Together with the propensity toward exploration this may explain low copying fidelity, seeing as kea are likely to be very interested in the potential affordances of objects (Diamond and Bond 1999; Huber et al*.*, 2001) and hence in applying trial and error-based strategies. This predisposition arguably is opposed to any tendency to reproduce demonstrated actions even in the first instance and instead would suggest a stronger likelihood that kea devise their own behavioural strategies from the get go, i.e. engage in emulation learning (Tomasello 1998). Previous studies indicate exactly this propensity and, given the aforementioned profile, provide a very probable interpretation of kea behaviour and social learning mechanisms. Hence, kea may have a predisposition towards emulation over imitation in their response to any given demonstration, based on their natural feeding ecology. Alternatively, kea may be flexible in applying any one social learning mechanism depending on the task. This would mean that the composition and complexity of the task guides the strategy applied. For instance, dogs have illustrated to apply inferential selective imitation in a rod-pulling task (Range et al. 2007) and in 2014 Kuczaj and Eskelinen could show that dolphins not only differentiated what to imitate from whom (i.e. individuals and context-dependent) but also that calves selectively only copied behaviours that were either novel or more complex than their own. This would mean that indiscriminate imitation might not be the sole strategy in social learning species but rather that some species may apply imitation selectively dependent on the “behavioral context, novelty of the behaviour, (and) significance of the model” (Kuczaj and Eskelinen 2014: 232).

One limitation of the study at hand may be a potential selection bias that was introduced through the test setup, as individuals were selected for the test groups that were tolerant towards the observation tunnel (see *Subjects* section). This might have inadvertently introduced a STRANGE-related bias (Webster & Rutz, 2020). Hence, a selection bias towards those individuals that were bolder and more exploratory and, therefore, could easily be trained to stay inside the observation tunnel. Subsequently, those individuals that were faster, in general, may have been selected for the test groups, hence, explaining any differences we see in the experimental treatment. The significant difference between the sexes in all scores, with the males performing more quickly than the females can potentially also be explained as an artefact of the test set-up (replication study) with four high-ranking males as demonstrators and an unbalanced male–female ratio across experimental groups (the test group had more males and the control group more females). To that effect, the selection bias may have amplified a known aspect of the social structure of kea. Diamond and Bond suggested that male kea are bolder and show more exploratory behaviour than females (Diamond and Bond 1991, 1999). Past experience has shown that male kea are faster to respond in test settings, while females seem to be more tolerant towards waiting, i.e. more patient. For instance, tested in a triadic cooperation task, male kea were faster to approach partners to initiate cooperation, however, females were better at waiting for the second partner to collectively solve the task (Schwing et al. 2020). An additional constraint on female participation may have been the breeding season, which leaves females less motivated to participate in testing. The combination of a selection bias (resulting in uneven sex ratio within the experimental groups), sex difference in general motivation to participate, paired with the seasonal unavailability of females could explain the present results and should be closely monitored in future studies.

In conclusion, our results illustrated that exploration rates were high in those groups that received a demonstration, indicating motivational and attentional shifts and hence pointing toward social learning effects in observer birds during initial exposure to the task. The study offers a valuable contribution to the ever-growing literature on social learning, which is marked by a distinct lack of truly comparative studies, as noted by Galef and Whiten (2017). In fact, although there seems to be undisputed agreement on the value of replication studies the pressure to publish novel, positive (significant) results limit the availability of such studies, for a comprehensive review on the topic see Farrar et al. (2021).

In line with previous studies, our results suggest that kea have a tendency toward applying individual techniques to solving a task which opens the question of whether kea engage in motor imitation at all or if in contrast they favour emulation over motor imitation. Alternatively, the task may have been too simple, or the two actions (push and pull) too similar and hence not suitable to test for imitative behaviour. Corresponding with social learning behaviour, the low difficulty level of the task may not have been adequate to induce imitative behaviour as a response (Garcia-Nisa et al. 2023). Kea may apply imitation selectively (Kuczaj et al. 2012, 2014) depending on the complexity, context and novelty of the task which would require a test set-up conducive to this propensity. Future studies will have to focus on devising a task that will tip the balance in favour of the application of imitative behaviour. This could be achieved by increasing the overall complexity of the task, and therefore, making it less likely that kea depend on individual learning to solve the task. Alternatively, rewarding could be manipulated by introducing asymmetry in the pay-offs, in line with Aplin and colleagues (2017), to incentivise observers to apply imitative behaviour.

## Supplementary Information

Below is the link to the electronic supplementary material.

Supplementary file1 (PDF 411 KB)

Supplementary file2 (MP4 80851 KB)

Supplementary file3 (DOCX 518 KB)

Supplementary file4 (XLSX 46 KB)

Supplementary file5 (M4V 53219 KB)

Supplementary file6 (MP4 104830 KB)

## Data Availability

The r script and workspace (containing all data) is available at the Science Data Bank repository https://doi.org/10.57760/sciencedb.07974.

## References

[CR1] Akins CK, Zentall TR (1998). Imitation in Japanese quail: the role of reinforcement of demonstrator responding. Psychon Bull Rev.

[CR2] Akins CK, Klein ED, Zentall TR (2002). Imitative learning in Japanese quail (*Coturnix japonica*) using the bidirectional control procedure. Anim Learn Behav.

[CR3] Aplin LM, Sheldon BC, McElreath R (2017). Conformity does not perpetuate suboptimal traditions in a wild population of songbirds. Proc Natl Acad Sci USA.

[CR4] Auersperg AMI, Gajdon GK, Huber L (2009). Kea (*Nestor notabilis*) consider spatial relationships between objects in the support problem. Biol Let.

[CR5] Auersperg AMI, von Bayern AMP, Gajdon GK, Huber L, Kacelnik A (2011). Flexibility in problem solving and tool use of kea and new caledonian crows in a multi access box paradigm. PLoS ONE.

[CR6] Auersperg AMI, Szabo B, Von Bayern AMP, Kacelnik A (2012). Spontaneous innovation in tool manufacture and use in a Goffin’s cockatoo. Curr Biol.

[CR7] Auersperg AMI, von Bayern AMPI, Weber S, Szabadvari A, Bugnyar T, Kacelnik A (2014). Social transmission of tool use and tool manufacture in goffin cockatoos (Cacatua goffini). Proce Royal Soci: Biol Sci.

[CR10] Bates D, Mächler M, Bolker B, Walker S (2015). Fitting linear mixed-effects models using lme4. J Statist Soft..

[CR11] Bugnyar T, Huber L (1997). Push or pull: an experimental study on imitation in marmosets. Anim Behav.

[CR12] Call J (2001). Body imitation in an enculturated orangutan (*Pongo pygmaeus*). Cybernet Syst.

[CR13] Clay Z, Tennie C (2018). Is overimitation a uniquely human phenomenon?. Insight Human Child Comp Bonob.

[CR14] Custance DM, Bard KA, Whiten A (1995). Can young chimpanzees (*Pan Troglodytes*) imitate arbitrary actions? hayes & hayes (1952) revisited. Behaviour.

[CR15] Dawson BV, Foss BM (1965). Observational learning in budgerigars. Anim Behav.

[CR16] Diamond J, Bond AB (1991). Social behaviour and the ontogeny of foraging in the kea (*Nestor notabilis*). Ethology.

[CR17] Diamond J, Bond A (1999). Kea, bird of paradox: the evolution and behavior of a New Zealand parrot.

[CR18] Farrar BG, Ostojić L, Clayton NS (2021). The hidden side of animal cognition research: Scientists’ attitudes toward bias, replicability and scientific practice. PLoS ONE.

[CR19] Fawcett TW, Skinner AMJJ, Goldsmith AR (2002). A test of imitative learning in starlings using a two-action method with an enhanced ghost control. Anim Behav.

[CR20] Forstmeier W, Schielzeth H (2011). Cryptic multiple hypotheses testing in linear models: overestimated effect sizes and the winner’s curse. Behav Ecol Sociobiol.

[CR22] Fugazza C, Pogány Á, Miklósi Á (2015). Do as I Did Long-term memory of imitative actions in dogs (Canis familiaris). Animal Cognit.

[CR23] Funk MS (2002). Problem solving skills in young yellow-crowned parakeets (Cyanoramphus auriceps). Anim Cogn.

[CR24] Gajdon GK, Fijn N, Huber L (2004). Testing social learning in a wild mountain parrot, the kea (*Nestor notabilis*). Anim Learn Behav.

[CR25] Gajdon GK, Fijn N, Huber L (2006). Limited spread of innovation in a wild parrot, the kea (Nestor notabilis). Anim Cogn.

[CR26] Gajdon GK, Ortner TM, Wolf CC, Huber L (2013). How to solve a mechanical problem: The relevance of visible and unobservable functionality for kea. Anim Cogn.

[CR03] Galef BG, Whiten A (2017) The comparative psychology of social learning. In: Call J, Burghardt GM, Pepperberg IM, Snowdon CT, Zentall T (eds) APA handbook of comparative psychology: Perception, learning, and cognition. American Psychological Association, pp 411–439. 10.1037/0000012-019

[CR02] Galef BG, Manzig LA, Field RM (1986). Imitation learning in budgerigars: Dawson and Foss (1965) revisited. Behav Process.

[CR27] Garcia-Nisa I, Evans C, Kendal RL (2023). The influence of task difficulty, social tolerance and model success on social learning in Barbary macaques. Sci Rep.

[CR29] Greco BJ, Brown TK, Andrews JRM, Swaisgood RR, Caine NG (2013). Social learning in captive African elephants (Loxodonta africana africana). Anim Cogn.

[CR31] Heyes CM (1994). Social learning in animals: categories and mechanisms. Biol Rev Camb Philos Soc.

[CR32] Heyes CM (2001). Causes and consequences of imitation. Trends Cogn Sci.

[CR34] Heyes CM, Dawson GR (1994). A demonstration of observational learning in rats using a bidirectional control. Quart J Exper Psychol Sect.

[CR35] Heyes C, Saggerson A (2002). Testing for imitative and nonimitative social learning in the budgerigar using a two-object/two-action test. Ani Behav..

[CR36] Heyes CM, Jaldow E, Dawson GR (1994). Imitation in rats: conditions of occurrence in a bidirectional control paradigm. Learn Motiv.

[CR37] Hoppitt W, Laland KN (2013) Social Learning. An Introduction to Mechanisms, Methods, and Models. In: Princeton NJ (eds): Princeton University Press. 10.1515/9781400846504

[CR38] Horner V, Whiten A (2005). Causal knowledge and imitation/emulation switching in chimpanzees (*Pan troglodytes*) and children (*Homo sapiens*). Anim Cogn.

[CR39] Huber L (2002). Clever birds: Keas learn through observation. Interpret Bird Bull.

[CR40] Huber L, Gajdon GK (2006). Technical intelligence in animals: The kea model. Ani Cognit.

[CR01] Huber L, Rechberger S, Taborsky M (2001). Social learning affects object exploration and manipulation in keas. Nestor notabilis. Anim Behav.

[CR41] Huber L, Range F, Voelkl B, Szucsich A, Viranyi Z, Miklosi A (2009). The evolution of imitation: what do the capacities of nonhuman animals tell us about the mechanisms of imitation?. Philosop Transact Royal Soc.

[CR43] Izawa EI, Watanabe S (2011). Observational learning in the large-billed crow (*Corvus macrorhynchos*): Effect of demonstrator-observer dominance relationship. Interact Stud.

[CR44] Kuczaj SA, Eskelinen HC (2014). Why do dolphins play*?*. An Behav Cognit..

[CR45] Kuczaj SA, Yeater D, Highfill L (2012). How selective is social learning in dolphins?. Int J Comparat Psychol.

[CR46] Lefebvre L (2000) Feeding innovations and their cultural transmission in birds. In Heyes, C.M. & Huber, L. (Eds) (2000). The evolution of cognition (pp. 311–328). Cambridge, MA: MIT Press.

[CR49] Mettke-Hofmann C, Winkler H, Leisler B (2002). The significance of ecological factors for exploration and neophobia in parrots. Ethology.

[CR50] Mitchell CJ, Heyes CM, Gardner MR, Dawson GR (2018). Limitations of a bidirectional control procedure for the investigation of imitation in rats: odour cues on the manipulandum. Quart J Exper Psychol Sect..

[CR51] Miyata H, Gajdon GK, Huber L, Fujita K (2011). How do keas (*Nestor notabilis*) solve artificial-fruit problems with multiple locks?. Anim Cogn.

[CR52] Moore BR (1992). Avian Movement Imitation and a New Form of Mimicry: Tracing the Evolution of a Complex Form of Learning. Behaviour.

[CR53] Nguyen NH, Klein ED, Zentall TR (2005). Imitation of a two-action sequence by pigeons. Psychon Bull Rev.

[CR54] Nielson M, Susianto EWE, Håkansson J (2010). Failure to find over-imitation in captive orangutans (Pongo pygmaeus): Implications for our understanding of cross-generation information transfer. Developmental Psychology.

[CR56] O’Hara M, Huber L, Gajdon GK (2015). The advantage of objects over images in discrimination and reversal learning by kea, *Nestor notabilis*. Anim Behav.

[CR57] O’Hara M, Schwing R, Federspiel I, Gajdon GK, Huber L (2016). Reasoning by exclusion in the kea (*Nestor notabilis*). Anim Cogn.

[CR58] Pepperberg IM, Funk MS (1990). Object permanence in four species of psittacine birds: An African Grey parrot (*Psittacus erithacus*), an Illiger mini macaw (*Ara maracana*), a parakeet (*Melopsittacus undulatus*), and a cockatiel (*Nymphicus hollandicus*). Anim Learn Behav.

[CR60] Range F, Viranyi Z, Huber L (2007). Selective imitation in domestic dogs. Curr Biol.

[CR61] Richardson PJ, Boyd R (2000) Climate, culture, and the evolution of cognition. In Heyes, C.M. & Huber, L. (Eds) (2000). The evolution of cognition (pp. 329–346).Cambridge, MA: MIT Press.

[CR64] Schwing R, Reuillon L, Conrad M, Noë R, Huber L (2020). Paying attention pays off: Kea improve in loose-string cooperation by attending to partner. Ethology.

[CR65] Thorpe, W. H. (1963). Learning and instinct in animals*.* (eds). London: Methuen.

[CR66] Tomasello M (1998). Emulation learning and cultural learning. Behav Brain Sci.

[CR67] Tomasello M, Gust D, Frost GT (1989). A longitudinal investigation of gestural communication in young chimpanzees. Primates.

[CR68] Tomasello M, Savage-Rumbaugh S, Kruger AC (1993). Imitative learning of actions on objects by children, chimpanzees, and enculturated chimpanzees. Child Dev.

[CR69] Topál J, Byrne RW, Miklósi Á, Csányi V (2006). Reproducing human actions and action sequences: “Do as I do!” in a dog. Anim Cogn.

[CR70] Veit A, Weißhaupt S, Bruat A, Wondrak M, Huber L (2023). Emulative learning of a two-step task in free-ranging domestic pigs. Anim Cogn.

[CR71] Voelkl B, Huber L (2000). True imitation in marmosets. Anim Behav.

[CR04] Webster MM, Rutz C (2020). How STRANGE are your study animals?. Nature.

[CR72] Werdenich D, Huber L (2006). A case of quick problem solving in birds: string pulling in keas. Nestor Not Ani Behav.

[CR73] Whiten A, Ham R (1992). On the nature and evolution of imitation in the animal kingdom: Reappraisal of a century of research. Adv Study Behav.

[CR74] Whiten A, McGuigan N, Marshall-Pescini S, Hopper LM (2009). Emulation, imitation, over-imitation and the scope of culture for child and chimpanzee. Philos Trans Royal Soc Biol Sci.

[CR75] Zajonc RB, Sales SM (1966). Social facilitation of dominant and subordinate responses. J Exp Soc Psychol.

[CR76] Zentall TR (2006). Imitation: definitions, evidence, and mechanisms. Ani Cognit..

