# Correction: Two-action task, testing imitative social learning in kea (*Nestor notabilis*)

**DOI:** 10.1007/s10071-023-01803-z

**Published:** 2023-06-21

**Authors:** Elisabeth Suwandschieff, Amelia Wein, Remco Folkertsma, Thomas Bugnyar, Ludwig Huber, Raoul Schwing

**Affiliations:** 1 Research Station Haidlhof, Comparative Cognition, Messerli Research Institute, University of Veterinary Medicine Vienna, Vienna, Austria; 2 Platform Bioinformatics and Biostatistics, University of Veterinary Medicine Vienna, Vienna, Austria; 3 Department of Cognitive Biology, University of Vienna, Vienna, Austria

**Correction: Animal Cognition ** 10.1007/s10071-023-01788-9

Unfortunately the authors given name and family name were swapped and the correct names are given below. The affiliation "Research Station Haidlhof, Comparative Cognition, Messerli Research Institute, University of Veterinary Medicine Vienna, Austria" is added. The Electronic Supplementary Materials are added. Some references were missing the DOIs and are included which is given below. The original article has been corrected.

Elisabeth Suwandschieff.

Amelia Wein.

Remco Folkertsma.

Thomas Bugnyar.

Ludwig Huber.

Raoul Schwing.

## Supplementary Information

Below is the link to the electronic supplementary material.

Supplementary file1 (PDF 411 KB)

Supplementary file2 (PDF 517 KB)
