# Supplementary material for: Two-action task, testing imitative social learning in kea (Nestor notabilis)
Source: Anim Cogn. 2023 Jun 1;26(4):1395–408. doi: 10.1007/s10071-023-01788-9 (PMC10345029; doi:10.1007/s10071-023-01788-9)
Supplement: Supplementary file 1 — Supplementary file1 (PDF 411 KB) [file 10071_2023_1788_MOESM1_ESM.pdf]

Supplementary Materials for

Two-action task, testing imitative social learning in kea (*Nestor notabilis*)

Journal name: Animal Cognition

Authors: Suwandschieff Elisabeth[a*], Wein Amelia[a], Folkertsma Remco[a,b], Bugnyar Thomas[c], Huber Ludwig[a], Schwing Raoul[a]

Affiliations:

[a] Comparative Cognition, Messerli Research Institute, University of Veterinary Medicine Vienna, Vienna, Austria

[b] Platform Bioinformatics and Biostatistics, University of Veterinary Medicine Vienna, Vienna, Austria

[c] Department of Cognitive Biology, University of Vienna, Vienna, Austria

* Corresponding author: Elisabeth Suwandschieff, Elisabeth.suwandschieff@vetmeduni.ac.at

Included: Model descriptions and Figures 6 – 10 (continued consecutive numbering from main text): results colour choice, side choice, approach pace, response duration and removal latency plus references.

Excluding: Model output

Models general

The models were fitted in R (version 4.2.0) (R Core Team, 2022) using the functions "glmer" or "lmer" of the package lme4 (version 1.1-27.1) (Bates *et al.*, 2015) with the optimizer "bobyqa" with 100.000 iterations.

Model descriptions per variable and figures

Response variable: Removal response

Data: all sessions

We fitted a generalized linear mixed model (rr.full.wc) with binomial error structure and logit link function to analyse the effect of experimental group on removal response. We used the function "glmer" of the package lme4 (version 1.1-27.1) (Bates *et al.*, 2015) with the optimizer "bobyqa" with 100.000 iterations. As a binary response variable we included whether an individual pulled

(coded as 0) or pushed (coded as 1) the stopper. As test predictors we included experimental group; session number; trial number and all their interactions up to the third order. Age and sex were included as control predictors. Before being included in the model, covariates session number; trial number and age were z-transformed to ease model convergence and achieve easier interpretable model coefficients (Schielzeth, 2010). To account for repeated observations of the same individual as well as to avoid pseudo-replication we included the random intercept effects of individual. Additionally, to model day specific variation in motivation/mood we included a factor combining session number and individual ("sessionID") nested within individual. To avoid overconfident models and to keep the Type I error rate at the nominal level of 0.05 (Barr *et al.*, 2013; Schielzeth & Forstmeier, 2009), we included all possible identifiable random slopes of session number and trial number and their interaction in individual as well as the random slope of trial number in sessionID. Including random slopes allows for its predictors effect to vary across levels of the random intercepts effect. For instance the random slope of trial number within sessionID takes into account the possibility that the removal response might vary in a trial number-related fashion within sessionID (ie depending on an individuals mood the removal response might differ across trial number). We removed correlations between random slopes and intercepts from the model when they were in part unidentifiable (with absolute correlation parameters estimated as 1) (Matuschek *et al.*, 2017).

After fitting the full model we confirmed that the none of the model assumptions were violated and assessed model stability. We verified absence of collinearity by calculating the Variance Inflation Factor (VIF) using the R package "car" version 3.0-12 (Fox & Weisberg, 2019). We visually inspected the best linear unbiased predictors (BLUPs) per level of the random effects were approximately normally distributed (Baayen, 2008; Harrison *et al.*, 2018).

We assessed model stability with regard to the model estimates, by comparing the estimates from the model including all data with estimates obtained from models in which the levels of random effects were excluded one at a time (Nieuwenhuis *et al.*, 2012). Unfortunately the model was of rather poor stability (see Online Resource 2, tab rem.resp_s17), which was obviously caused by only a limited number of observations where the response was "push", resulting in instance of complete separation (Gelman & Hill 2006). However, this does not restrain from inference on the relevant terms of interest in the model, if any it would make the *p*-values more conservative.

We compared the full model with all terms included, to a null model lacking the key terms of interest (experimental group; session number; trial number and all their interactions up to the third order) to avoid 'cryptic multiple testing' (Forstmeier & Schielzeth, 2011). We found no effect of the test predictors experimental group; session number; trial number or their interaction (χ^2 = 6.630, df = 7, P = 0.468). We calculated confidence intervals for the model estimates by applying the function 'bootMer' of the package 'lme4', using 1,000 parametric bootstraps.

Response variable: Stopper colour

Data: all sessions

Analysis of choice of stopper colour was very similar to the analysis for removal response (see Online Resource 2, c.full.wc). Choice of stopper colour was modelled using the test predictors experimental group, session and trial, as well as all their interactions up until the third order. As control predictors we included age and sex. The full-null model comparison revealed that overall, the test predictors had an impact (χ^2 = 22.974, df = 7, P = 0.0017) on the outcome variable, and there was a small but significant interaction between experimental group, session and trial. The probability to choose white increased slightly over sessions, the effect of trial within session was constant for birds in the control group but increased slightly for observer birds (test group) which became more pronounced in later sessions. We reduced the model further to inspect the main effect of experimental group. This revealed that there was no difference between experimental groups concerning colour choice (χ^2 = 2.041, df = 1, P = 0.153), see Figure 6. Both groups had a minimal preference for white and there was a high variation in the individual choosing patterns across sessions and trials.

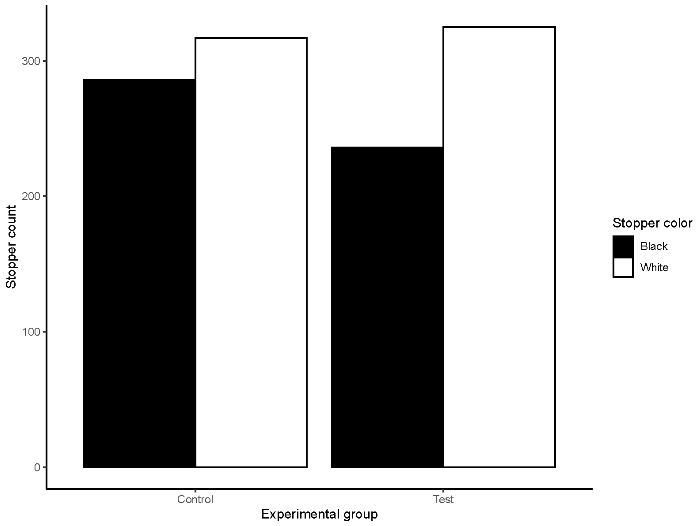

Figure 6 Mean of colour choice (black vs. white) separated into the experimental group experimental

To check whether individuals adopted a preference for the demonstrated colour. We ran a separate model on a subset of the data including only observer birds using only assigned (i.e. demonstrated) colour as test predictor and subject as random effect This revealed no preference for the demonstrated colour (χ^2 = 0.179, df = 1, P = 0.67), see Online Resource 2.Response variable: Stopper side

Data: all sessions

Using an identical model structure as in stopper colour in the explanatory part of the model, and only differing in the response part (now side) we analysed the stopper side choice. There was no difference between experimental groups concerning the side choice (χ^2 = 0.125, df = 1, P =

0.72). Although there was a clear propensity towards choosing the left side (see Figure 7), this was the same across experimental groups. The left side was the closest to the individuals of both experimental groups when entering the compartment (Online Resource 2, s.full.wc).

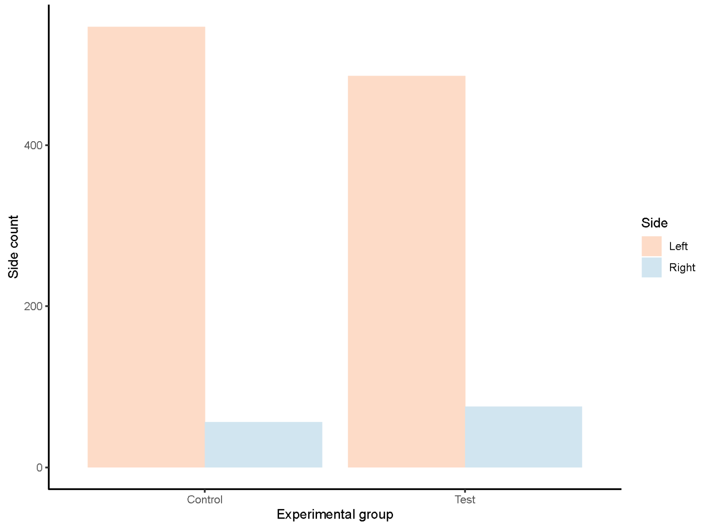

Figure 7 Mean of side choice (left vs. right) separated into the experimental group.

Response variable: Approach pace

Data: all sessions, session one only, session two-seven

We fitted a linear mixed model (ap.full.wc, Online Resource 2) to analyse the effect of experimental group on approach pace (log transformed to improve fit for linear model assumptions). We used the function “lmer” of the package lme4 (version 1.1-27.1) (Bates *et al.*, 2015) with the optimizer “bobyqa” with 100.000 iterations. We compared the full model to a null model lacking the effects of experimental group, trial.nr and their interaction. Because the full-null model comparison did not reveal an effect of the test predictors experimental group; session number; trial number or their interaction (χ^2 = 9.775, df = 7, P = 0.202) we did not test the individual fixed effects.

Data: session 1 only and session 2-7

The linear mixed model for session one (ap.s1.full.wc) included experimental group, trial number and their interaction as test predictors. As control predictors we included age and sex. The covariates trial.nr and age were z-transformed before including them into the model. To account for repeated observations of the same individuals we included Subject as a random intercept effect. To keep the Type I error rate at the nominal level of 0.05 we included the random slope of trial.nr within Subject. We included the correlation between random intercept and random slope, but decided to remove this if it was unidentifiable (indicated by absolute correlation parameters estimated as 1). Just as for model rr.full.wc, we checked model issues, models assumptions and model stability.

The linear mixed model for session two-seven (ap.s27.full.wc) included experimental group; session number; trial number and all their interactions up to the third order as test predictors. Age and sex were included as control predictors. Subject and SessionID were included as random intercept effects and included were all possible identifiable random slopes of session, trial and their interaction. We included the correlations between random intercepts and random slopes, but decided to remove these if they were in part unidentifiable (indicated by absolute correlation parameters estimated as 1). We checked model issues, models assumptions and model stability. The full model was compared to a model lacking the test predictors, which revealed no effect of the test predictors (χ^2 = 6.828, df = 7, P = 0.447).

None of the models revealed an effect of the test predictors (all sessions: χ^2 = 9.775, df = 7, P = 0.202; only session one: χ^2 = 3.2523, df = 3, P = 0.318; only session two-seven: χ^2 = 6.828, df = 7, P = 0.447), see Figure 8.

A

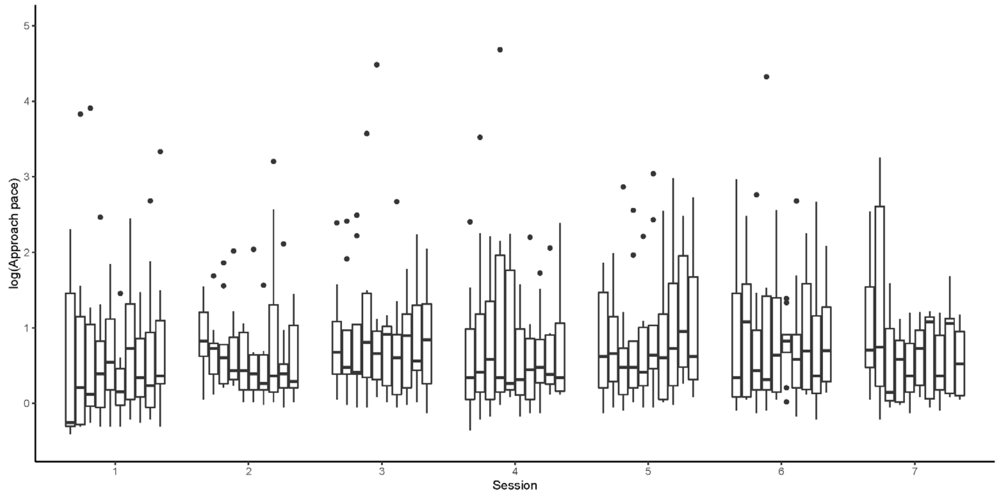

B

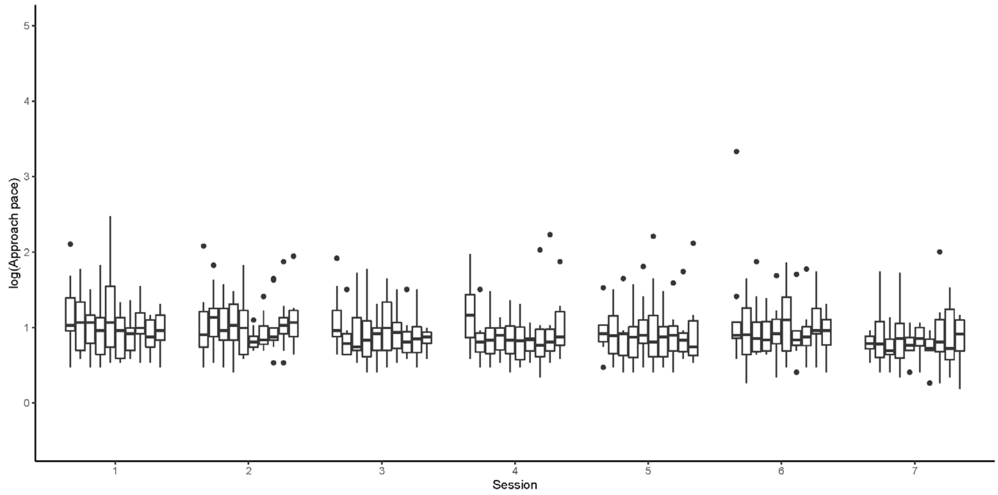

Figure 8 Log of approach pace in log seconds across all trials and test sessions, (A) control group and (B) test group. Boxplots show the median (solid line), 25th–75th percentile (box) and the largest and smallest value (whiskers). Dots

reflects outliers. Note that the figures show the raw data and does not represent the fitted model with log (approach pace) as a response. Full-null model comparison χ^2 = 9.775, df = 7, P = 0.202

Response variable: Response duration

Data: all sessions

We fitted a linear mixed model (rd.full.wc) to analyse the effect of experimental group on response duration (log transformed to improve fit for linear model assumptions). We used the function "lmer" of the package lme4 (version 1.1-27.1) (Bates *et al.*, 2015) with the optimizer "bobyqa" with 100.000 iterations. The full model and the null model were identical to the model for removal response (rr.full.wc) with respect to the fixed effect as well as the random effects (for details for all models see Online Resource 2 , tab: model_overview). To check for model issues, assumptions and model stability we performed the same steps as described above for model rr.full.wc Because the full-null model comparison revealed an effect of the test predictors experimental group; session number; trial number or their interaction (χ^2 = 24.448, df = 7, *P* = 0.0010) we tested the individual fixed effects to achieve informative estimates of the fixed effects terms. We did so by reducing model complexity and dropping non-significant interactions, from higher order to lower order terms, from the model one at a time and compare the simpler with the more complex model utilizing likelihood ratio tests (see Online Resource 2 , tab rd.full.wc). This revealed a significant interaction between session and trial (χ^2 = 11.488, df = 1,*P* = 0.0007), but there was no difference between experimental groups concerning the response duration across the ten trials and seven sessions (χ^2 = 0.325, df = 1,*P* = 0.569), see Figure 9.

However, the analysis did not control for the effect of removal latency in response duration. To verify that any significant results we have found in response duration were not a remnant of removal latency we therefore ran an additional model post-hoc including removal latency as a predictor, (rd.alt.full.wc). The full-null model comparison (null model excluding experimental group, trial, session and their interaction) did not result in a significant difference (χ^2 = 4.587, df = 7, *P* = 0.71). Hence, when controlling for removal latency, there was no clear influence of the predictors experimental group, trial, session, or any of their interactions on the response duration. Therefore, the analysis of session 1 *versus* session 2-7 was conducted controlling for removal latency as a predictor.

Data: session 1 only and session 2-7

The linear mixed model for session one (rd.alt.s1.full.wc) included experimental group, trial number and their interaction as test predictors. As control predictors we included age, sex and removal latency. The covariate removal latency was log-transformed and trial.nr, age and the log-transformed removal latency were z-transformed before including them into the model. To account for repeated observations of the same individuals we included Subject as a random intercept effect. To keep the Type I error rate at the nominal level of 0.05 we included the random

slope of trial.nr within Subject. We included the correlation between random intercept and random slope, but decided to remove this if it was unidentifiable (indicated by absolute correlation parameters estimated as 1). Just as for model 1_rem.resp, we checked model issues, models assumptions and model stability.

We compared the full model to a null model lacking the effects of experimental group, trial.nr and their interaction, which revealed a marginally significant effect ($\chi 2$ = 6.808, df = 3, P = 0.078). We consider this to provide evidence for an effect of the test predictors on the response, and thus we tested the individual effects as described above for model rr.full.wc. The analysis illustrated that there was indeed a significant effect of experimental group and trial number in session one of response duration (χ^2 = 6.105, df = 1, P = 0.014). Observer birds decreased their response duration over trials in session one, while in contrast, control group birds slightly increased in their response duration over trials in session one.

The linear mixed model for session two-seven (rd.alt.s27.full.wc) included experimental group; session number; trial number and all their interactions up to the third order as test predictors. age, sex and removal latency were included as control predictors, as above all covariates were transformed. Subject and SessionID were included as random intercept effects and all possible identifiable random slopes were included. We included the correlations between random intercepts and random slopes, but decided to remove these if they were in part unidentifiable (indicated by absolute correlation parameters estimated as 1). We checked model issues, models assumptions and model stability. The full model was compared to a model lacking the test predictors, which revealed no effect of the test predictors ($\chi 2$ = 6.641, df = 7, P = 0.4672).

A

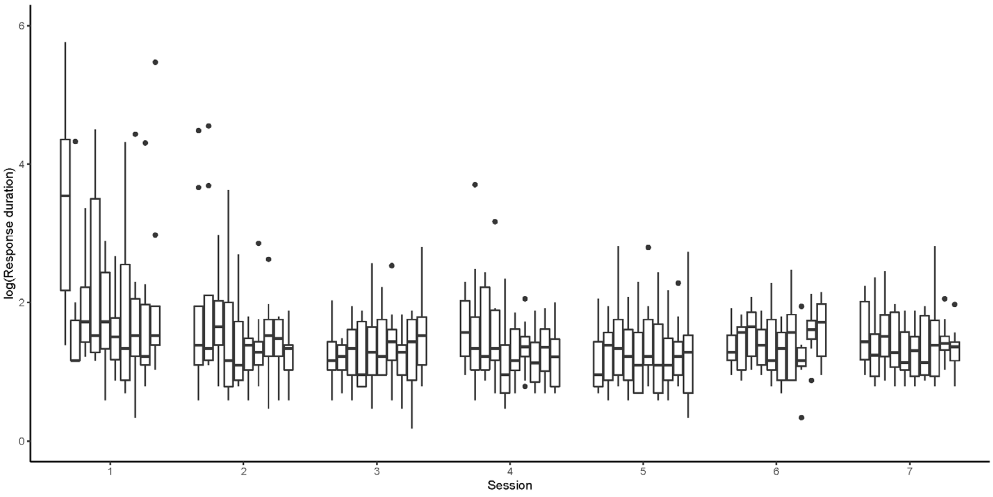

B

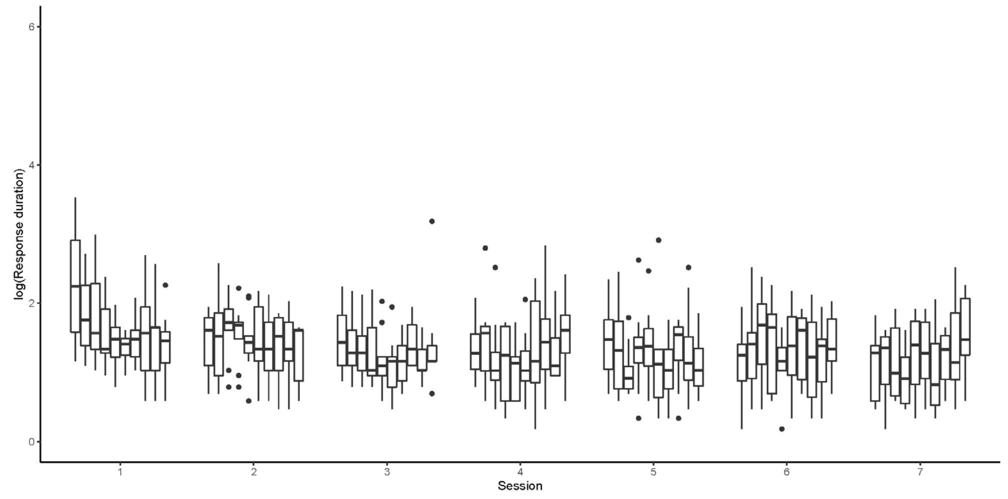

Figure 9 Log response duration in log seconds across all trials and test sessions, (A) control group and (B) test group. Boxplots show the median (solid line), 25th–75th percentile (box) and the largest and smallest value (whiskers). Dots reflects outliers. Note that the figures show the raw data and does not represent the fitted model with log (response duration) as a response. Full-null model comparison: $\chi 2$ = 24.448, df = 7, P = 0.0010

Response variable: Removal latency

Data: all sessions, session one only, session two-seven

We fitted several linear mixed models (rl.full.wc, rl.s1.full.wc, rl.s27.full.wc) to analyse the effect of experimental group on removal latency (log transformed to improve fit for linear model assumptions). The models were identical to the models as previously described for “Approach pace” with respect to the fixed effects and random effects (for details see Online Resource 2). We followed the same procedures with respect to model assumptions, model issues and stability of the model. We applied the same full-null model comparisons. The model including all observations (rl.full.wc) and including only session one (rl.s1.full.wc) revealed effects of the test predictors (χ^2 = 29.774, df = 7, P = 0.0001; and χ^2 = 21.579, df = 3, P = 0.0001, respectively), but the model including only session two to seven (rl.s27.full.wc) did not (χ^2 = 11.108, df = 7, P = 0.133).

For removal latency, the full-null model comparison revealed that overall the test predictors had an impact (χ^2 = 29. 774, df = 7, P = 0.0001) on the outcome variable. However, there was no difference between experimental groups concerning the latency to remove the stopper across the seven test sessions (χ^2 = 0.231, df = 1, P = 0.631). Both groups got faster in the removal latency at a similar rate, see Figure 10.

A

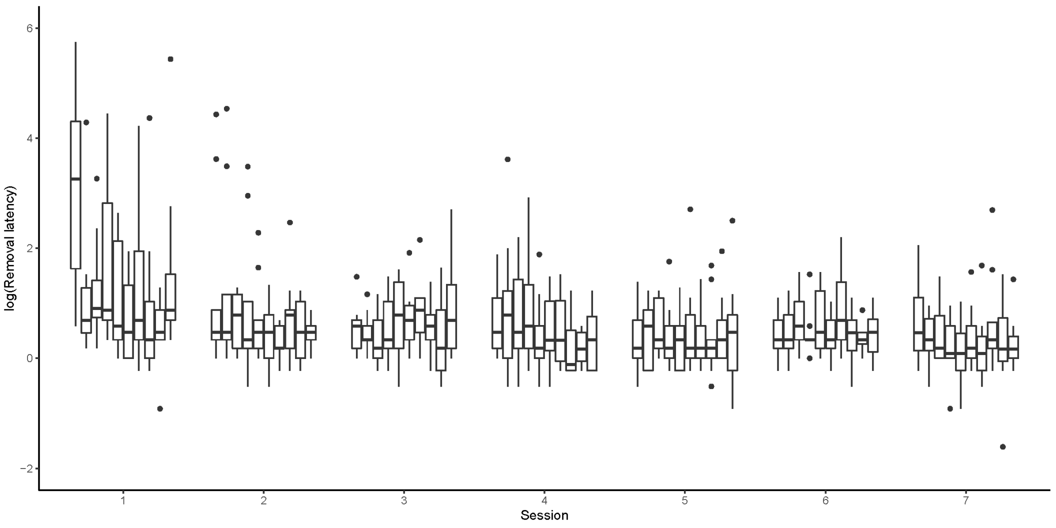

B

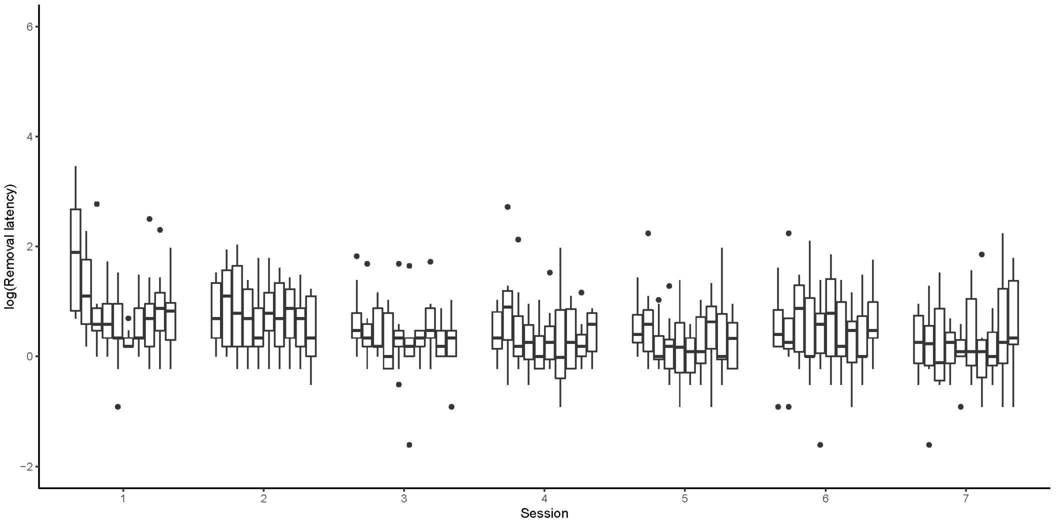

Figure 10 Log removal latency in log seconds across all trials and test sessions, (A) control group and (B) test group. Boxplots show the median (solid line), 25th–75th percentile (box) and the largest and smallest value (whiskers). Dots reflects outliers. Note that the figures show the raw data and does not represent the fitted model with log (removal latency) as a response. Full-null model comparison: χ^2 = 29.775, df = 6, P = 0.0001

<u>References</u>

Baayen, R. H. (2008). *Analyzing Linguistic Data: A Practical Introduction to Statistics using R*. Cambridge University Press. https://doi.org/DOI: 10.1017/CBO9780511801686

Barr, D. J., Levy, R., Scheepers, C., & Tily, H. J. (2013). Random effects structure for confirmatory hypothesis testing: Keep it maximal. *Journal of Memory and Language*, *68*(3), 10.1016/j.jml.2012.11.001. https://doi.org/10.1016/j.jml.2012.11.001

Bates, D., Mächler, M., Bolker, B., & Walker, S. (2015). Fitting Linear Mixed-Effects Models Using lme4. *Journal of Statistical Software*, *67*(1 SE-Articles), 1–48. https://doi.org/10.18637/jss.v067.i01

Forstmeier, W., & Schielzeth, H. (2011). Cryptic multiple hypotheses testing in linear models: overestimated effect sizes and the winner's curse. *Behavioral Ecology and Sociobiology*, *65*(1), 47–55. https://doi.org/10.1007/s00265-010-1038-5

Fox, J., & Weisberg, S. (2019). *An {R} Companion to Applied Regression* (Third). Sage. https://socialsciences.mcmaster.ca/jfox/Books/Companion/

Gelman, A., & Hill, J. (2006). Data analysis using regression and multilevel/hierarchical models. *Cambridge university press*. https://doi.org/10.1017/CBO9780511790942

Harrison, X. A., Donaldson, L., Correa-Cano, M. E., Evans, J., Fisher, D. N., Goodwin, C. E. D., Robinson, B. S., Hodgson, D. J., & Inger, R. (2018). A brief introduction to mixed effects modelling and multi-model inference in ecology. *PeerJ*, *6*, e4794. https://doi.org/10.7717/peerj.4794

Matuschek, H., Kliegl, R., Vasishth, S., Baayen, H., & Bates, D. (2017). Balancing Type I error and power in linear mixed models. *Journal of Memory and Language*, *94*, 305–315. https://doi.org/https://doi.org/10.1016/j.jml.2017.01.001

Nieuwenhuis, R., te Grotenhuis, M., & Pelzer, B. (2012). Influence.ME: Tools for detecting influential data in mixed effects models. *R Journal*, *4*(2), 38–47. https://doi.org/10.32614/rj-2012-011

R Core Team. (2022). *R: A Language and Environment for Statistical Computing*. https://www.r-project.org/

Schielzeth, H. (2010). Simple means to improve the interpretability of regression coefficients. *Methods in Ecology and Evolution*, *1*(2), 103–113. https://doi.org/https://doi.org/10.1111/j.2041-210X.2010.00012.x

Schielzeth, H., & Forstmeier, W. (2009). Conclusions beyond support: overconfident estimates in mixed models. *Behavioral Ecology : Official Journal of the International Society for Behavioral Ecology*, *20*(2), 416–420. https://doi.org/10.1093/beheco/arn145
