# Supplementary material for: Two-action task, testing imitative social learning in kea (Nestor notabilis)
Source: Anim Cogn. 2023 Jun 1;26(4):1395–408. doi: 10.1007/s10071-023-01788-9 (PMC10345029; doi:10.1007/s10071-023-01788-9)

Demonstrator Training: Phase I

- Stopper is in Position 1, which allows the subject to easily push it into the box
- Only one stopper is presented – the one which is the target color for that individual
- 1 session = 10 trials of stopper in Position 1
- Criteria is two consecutive sessions with no mistakes

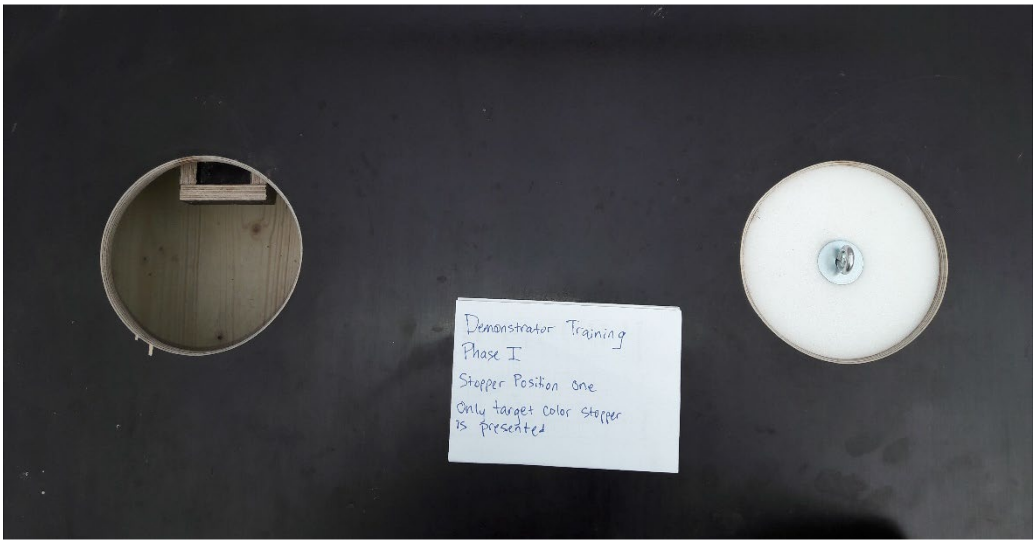

Fig 1 – box setup for demonstrator training phase I

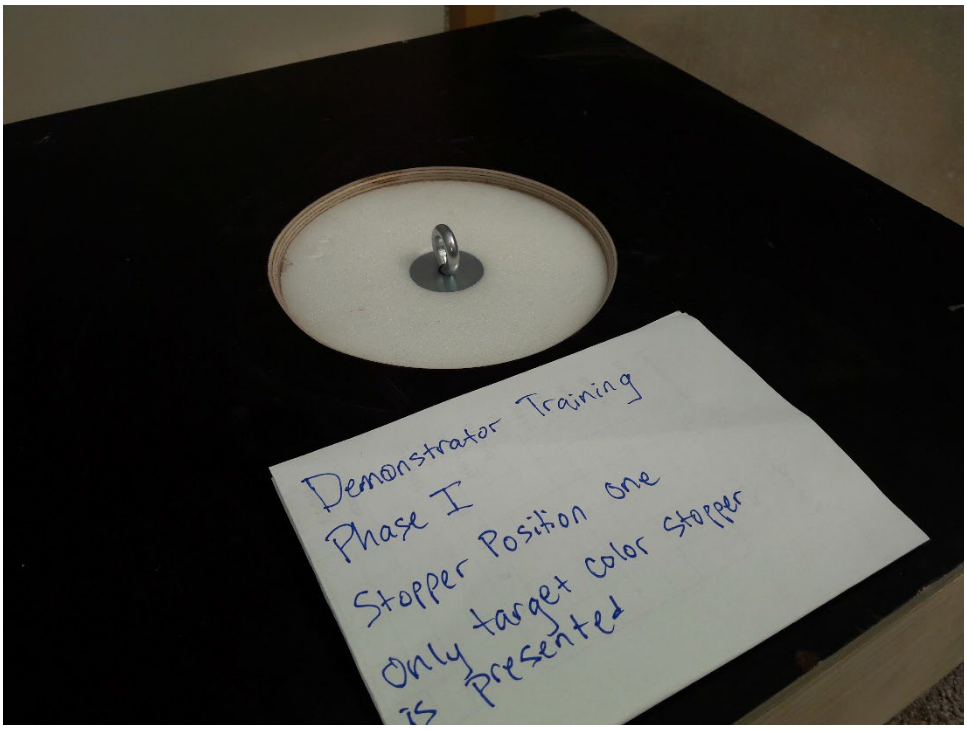

Fig 2 – close up of stopper position one

Demonstrator training: phase 2

Phase 2.1

- Stopper is in Position 1 for first five trials of the session, and Position Two for the second five trials of the session
- Stopper Position 2 – the stopper is flush with the box surface.
- Only the target stopper is presented
- Criteria to move on two Phase 2.2 is two consecutive sessions with no mistakes

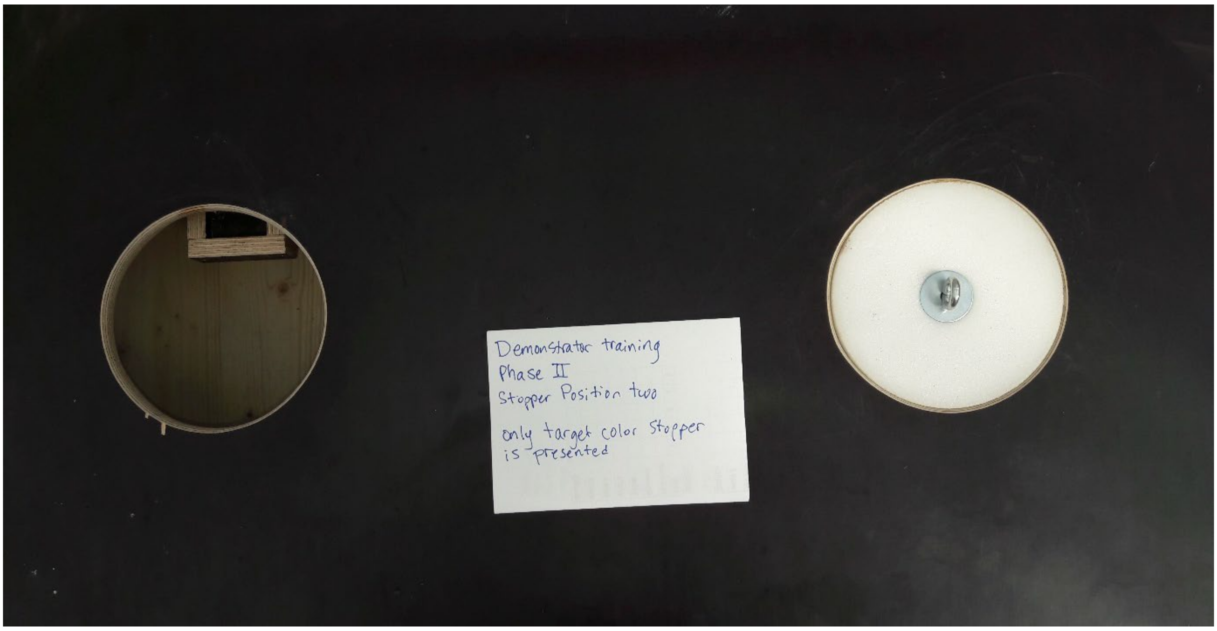

Fig 3 – box setup for demonstrator training phase 2

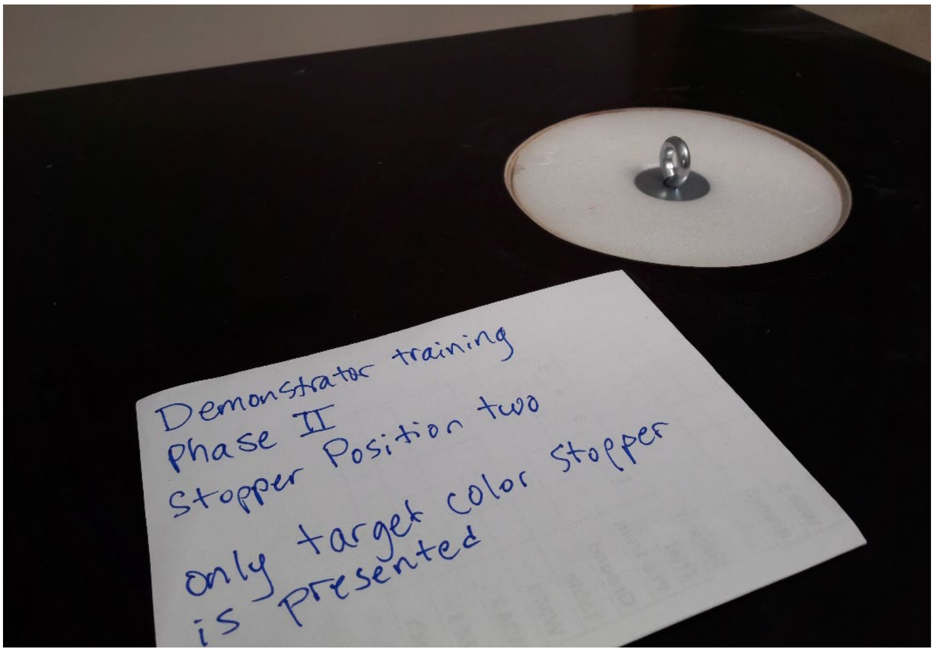

Fig 4 – close up of stopper Position 2, where the stopper is flush with the box.

Phase 2.2

- same as phase 2.1, but the stopper is in Position 2 for all 10 trials of the session.

Demonstrator training: phase 3

Phase 3.1

- Stopper is in Position 2 for first five trials of the session, and Position 3 for the second five trials of the session
- Stopper Position 3 – the stopper is pushed in all the way and is a few millimeters above the box surface.
- only the target stopper is presented
- Criteria to move on two Phase 3.2 is two consecutive sessions with no mistakes

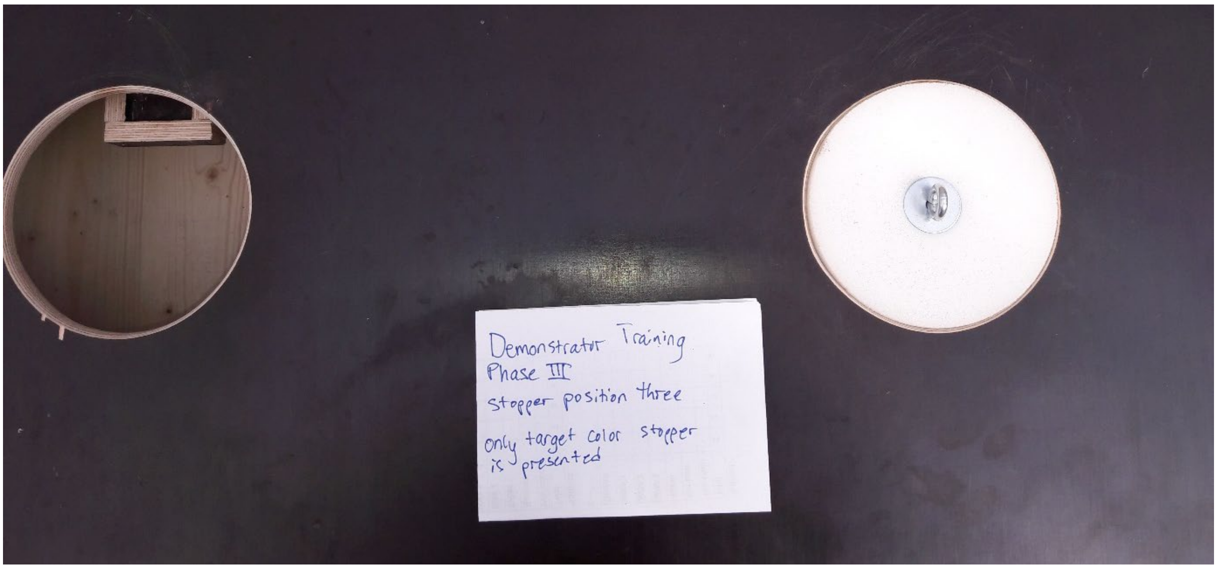

Fig 5 – Box setup for Phase 3

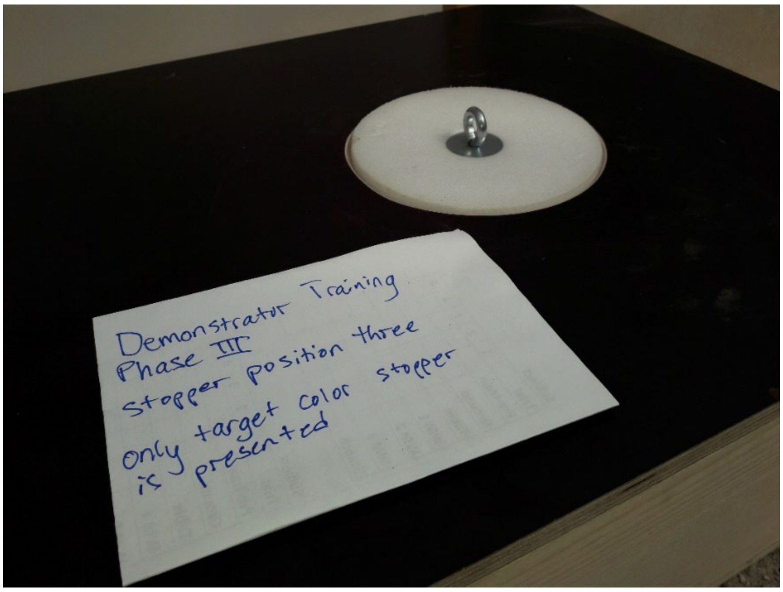

Fig 6 – close up of Stopper Position 3

Phase 3.2

- same as phase 3.1, but the stopper is in Position 3 for all 10 trials of the session.

Demonstrator training: phase 4

Phase 4.1

- Target stopper is in Position 2 for the first 5 trials of the session, Position 3 for the last five trials.
- Non-target stopper is in Position 3 for all trials.
- Criteria to complete training phase – 2 consecutive sessions with no mistakes
- **Repeat this training step as necessary.**

Phase 4.2

- Stoppers are in Position 3 for all trials
- Both stoppers are presented. Only the target stopper can be solved
- Criteria to complete training – 2 consecutive sessions with no mistakes.

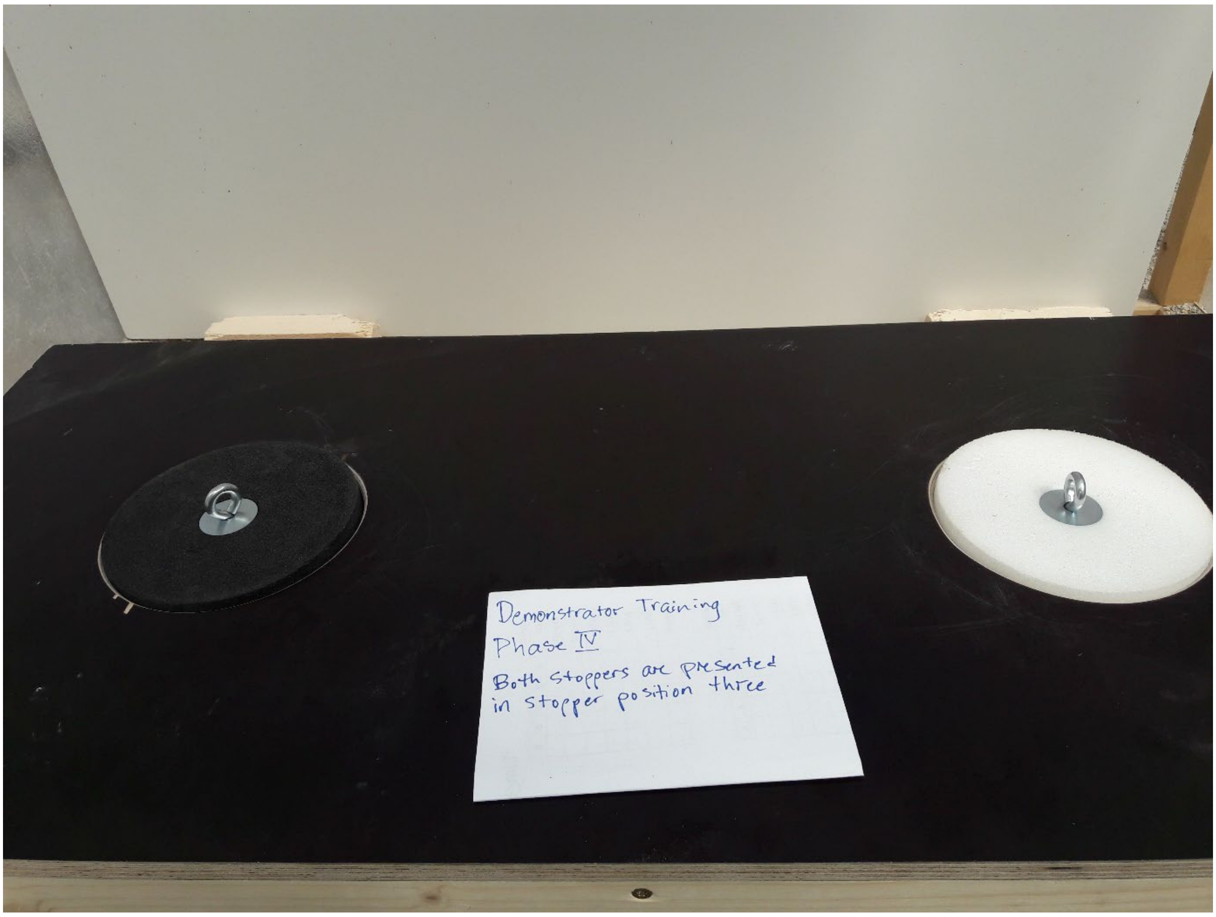

Supplement: Supplementary file 3 — Supplementary file3 (DOCX 518 KB) [file 10071_2023_1788_MOESM3_ESM.pdf]
